# QSPR analysis of amino acids for the family of Gourava indices

**DOI:** 10.1371/journal.pone.0319029

**Published:** 2025-04-29

**Authors:** Khadija Sarwar, Salma Kanwal, Asima Razzaque

**Affiliations:** 1 Department of Mathematics, Lahore College for Women University, Lahore, Pakistan; 2 Preparatory Year, Basic science, King Faisal University, Al-Ahsa, Saudi Arabia; 3 Department of Mathematics, College of Science, King Faisal University, Al-Ahsa, Saudi Arabia; Universidad Autonoma de Chihuahua, MEXICO

## Abstract

Amino acids are chemical molecules that act as the building blocks of proteins and perform critical functions in biological processes. Their two main functional groups, an amino group (-NH_2_) and a carboxyl group (-COOH) as well as a changeable side chain (R group) that controls the unique characteristics of each amino acid are what define them. Because they can serve as building blocks for a variety of macromolecules and support biological activities in a variety of ways, amino acids have a wide range of uses in biology, medicine, industry and nutrition.

Quantitative Structure-Activity/Property Relationships employ graph invariants to model physicochemical properties of substances. Topological indices are a reliable and computationally efficient technique to express molecular structures and properties, making them indispensable in theoretical and applied chemistry. Gourava indices are valuable mathematical tools that provide deeper insights into the topology and structure of networks and molecular graphs, resulting in improved decision-making and efficiency in research and applications. In this article, Gourva, hyper Gourava, alpha Gourava and gamma Gourava indices are presented and calculated. Curvilinear and multilinear regression models for predicting physicochemical characteristics of amino acids are analyzed.

## Introduction

Amino acids are the essential components of life. The body’s cells, tissues, and organs depend on these chemical substances for their proper construction, operation, and regulation. They are important in many biological activities, particularly protein synthesis, which is essential for practically all cellular functions. In pharmaceutical development and delivery, amino acids play an important role as therapeutic agents, excipients, and precursors. They are essential in treating a wide range of medical disorders due to their distinct chemical characteristics and biological roles. Aside from being active components, amino acids are also used as excipients in medication formulations. Glycine, for example, functions as a buffering agent to keep pharmaceutical goods stable and pH stable, whereas lysine and arginine improve the solubility of medications that are normally insoluble in water. Amino acids offer vital help in the field of metabolic disorders. In order to compensate for metabolic deficiencies, patients with hereditary disorders such as phenylketonuria (PKU) or maple syrup urine disease (MSUD) need to follow carefully monitored diets enriched with essential amino acids. A vital treatment for acute lymphoblastic leukemia (ALL) is also asparaginase, an enzyme that degrades the amino acid asparagine. Moreover, amino acids improve drug delivery mechanisms. To increase their solubility and absorption, some medications are conjugated with amino acids. To treat viral infections, for instance, valacyclovir, a prodrug of acyclovir coupled with valine, guarantees improved bioavailability and efficacy. The use of amino acids in prodrugs has improved features such as bioavailability, reduced toxicity, targeted delivery, and prevention of rapid metabolism [1]. Amino acids are useful in treatments for oxidative stress and anti-aging because of their antioxidant qualities. Ongoing studies on amino acid metabolism in personalized medicine present encouraging treatment options for neurological conditions, metabolic diseases, and cancer. For example, a lot of cancer treatments focus on the tumor cells’ reliance on particular amino acids, such as glutamine. This demonstrates their capacity to provide precise, focused therapies. Drug research relies heavily on natural compounds and derivatives, yet their low solubility and activity sometimes necessitate structural modifications. Amino acids are extremely soluble in water and have a diverse range of functions. Adding amino acids to natural goods can enhance their performance while reducing negative effects [2].

Chemical graph theory is a subfield of mathematical chemistry that uses graph theory to represent and analyze molecular structures [3]. In this technique, molecules are represented as graphs, with atoms as vertices and chemical bonds as edges. The topic focuses on understanding molecular features and behaviors using mathematical descriptions known as graph invariants. Chemical graph theory has a wide range of applications, including predicting physical and chemical properties in quantitative structure-activity/property relationships (QSAR/QSPR), modeling reaction mechanisms, and evaluating bigger molecular networks like metabolic pathways. NetworkX(Python), RDKit, and molecular modeling software are all examples of tools that use graph-theoretic approaches for these purposes. Research in this subject also extends to cutting-edge domains such as nanotechnology, where it aids in the study of materials like as carbon nanotubes and graphene, and drug discovery, where graph-based algorithms help anticipate molecular interactions. Furthermore, advances in artificial intelligence and machine learning are improving the predictive powers of chemical graph theory, making it an indispensable tool in modern chemistry and material science.

Topological indices are numerical values generated from a molecule’s graph representation and used as structural descriptors [4]. They are widely employed in chemical graph theory to forecast and simulate physicochemical attributes, biological activity, and other molecular characteristics [5]. These indices capture crucial characteristics of molecule geometry, connectivity, and branching, making them useful in applications like quantitative structure-property relationships (QSPR) and quantitative structure-activity relationships (QSAR). The chemical and biological sciences as well as engineering use quantitative structure–activity relationship models (QSAR models), which are regression or classification models [6,7]. The procedure entails gathering data on known compounds, calculating descriptors, and establishing correlations through regression or classification approaches. These models are tested using metrics, R^2^, RMSE, and cross-validation to ensure robustness and forecast accuracy. QSPR and QSAR allow for fast design and screening of novel compounds, reducing the requirement for experimental testing. They are frequently used in drug development, material research, and environmental risk assessment. Integrating QSPR with machine learning improves its predictive capacity, allowing for the discovery of new compounds with desired properties while minimizing reliance on time-consuming and expensive experimental approaches. This method speeds up the medication design process, identifies potential concerns early on, and contributes to the efficient development of safer and more effective therapies.

In chemical graph theory, the Randi$c^{\prime }$ index is among the well-known degree based topological indices. Since its introduction, it has been thoroughly researched and applied to represent the characteristics that define particular molecular structures. The Randi$c^{\prime }$ index is frequently applied in chemistry excercises, particularly in QSPR/QSAR studies. Randi$c^{\prime }$ [8] proposed the Randi$c^{\prime }$ index, which is referred as


$$\begin{array}{ccc} R_{\alpha }(G)=\underset{xy\in E(G)}{\sum }{\left(d_{G}(x)d_{G}(y)\right)}^{\alpha } & & \end{array}$$ (1)

By modifying the Randic′ index concept, Zhou and Trinajsti$c^{\prime }$ [9] created a new index known as the General sum-connectivity index, which has the following definition.


$$\begin{array}{ccc} \chi_{\alpha }(G)=\underset{xy\in E(G)}{\sum }{\left(d_{G}(x)+d_{G}(y)\right)}^{\alpha } & & \end{array}$$ (2)

Over thirty years ago, Gutman and Trinajsti$c^{\prime }$ [10] explored the structure-dependency of total *π*-electron energy and provided these approximated expressions.


$$\begin{array}{ccc} M_{1}(G)=\underset{xy\in E(G)}{\sum }(d_{G}(x)+d_{G}(y)) & & \end{array}$$ (3)

$$\begin{array}{ccc} M_{2}(G)=\underset{xy\in E(G)}{\sum }(d_{G}(x)d_{G}(y)) & & \end{array}$$ (4)

V. R. Kulli [11], driven by the criteria of the Zagreb indices and their potential applications, presented the first and second Gourava indices as follows.


$$\begin{array}{ccc} GO_{1}(G)=\underset{xy\in E(G)}{\sum }(d(x)+d(y)+d(x)d(y)) & & \end{array}$$ (5)

$$\begin{array}{ccc} GO_{2}(G)=\underset{xy\in E(G)}{\sum }(d(x)+d(y))(d(x)d(y)) & & \end{array}$$ (6)

V.R. Kulii [12] presented first and second hyper Gourava indices.


$$\begin{array}{ccc} HGO_{1}(G)=\underset{xy\in E(G)}{\sum }[(d(x)+d(y)+d(x)d(y){]}^{2} & & \end{array}$$ (7)

$$\begin{array}{ccc} HGO_{2}(G)=\underset{xy\in E(G)}{\sum }[(d(x)+d(y)(d(x)d(y)){]}^{2} & & \end{array}$$ (8)

First and second Alpha Gourava indices are defined as


$$\begin{array}{ccc} AGO_{1}(G)=\underset{xy\in E(G)}{\sum }(d{\left(x\right)}^{2}+d{\left(y\right)}^{2}+d(x)d(y)) & & \end{array}$$ (9)

$$\begin{array}{ccc} AGO_{2}(G)=\underset{xy\in E(G)}{\sum }(d{\left(x\right)}^{2}+d{\left(y\right)}^{2}(d(x)d(y)) & & \end{array}$$ (10)

First and second Gamma Gourava indices [13,14] are defined as


$$\begin{array}{ccc} GGO_{1}(G)=\underset{xy\in E(G)}{\sum }(d{\left(x\right)}^{3}+d{\left(y\right)}^{3}+d(x)d(y)) & & \end{array}$$ (11)

$$\begin{array}{ccc} GGO_{2}(G)=\underset{xy\in E(G)}{\sum }(d{\left(x\right)}^{3}+d{\left(y\right)}^{3})(d(x)d(y)) & & \end{array}$$ (12)

In data science, networks and graphs are widely used to depict relationships, dependencies, and interactions. Gourava indices are used to examine the structural complexity and similarity of such networks for a wide range of applications. The computation of Gourava indices allows researchers to determine how changes to the chemical structure, such as the addition or removal of atoms or bonds, alter the compound’s toxicity, activity, or pharmaceutical effectiveness. Gourava and hyper-Gourava indices are chemically applicable [15].

Quantitative Structure-Property Relationship (QSPR) and Quantitative Structure-Activity Relationship (QSAR) are computational methodologies for predicting molecular properties and biological activities using structural descriptors. Safari and Shafiei studied the derivatives of amino acids using topological indices and analyzed their thermodynamic properties [16]. Another study of thermodynamic properties of some monocarboxylic acids using multiple regression model was presented by Havare [17]. The study by H. M. Nagesh [18] used temperature-based topological indices (sum connectivity, product connectivity, F-temperature, and symmetric division) to predict thermodynamic properties of monocarboxylic acids, including enthalpies of formation, combustion and vaporization. To analyze the correlation between thermodynamic parameters and topological indices, a curvilinear regression model is used with linear, quadratic, and cubic equations. At the end a comparison was made. The study in [19] utilized three degree-based topological indices to predict six physicochemical characteristics of 16 alkaloid compounds. Topological indices and experimental values serve as inputs for linear and quadratic regression models. All indices have substantial correlations and p-values, indicating the results’ validity and utility. Inoue et al. in [20] studied the different acyclovir ointments and the results obtained imply that the physicochemical characteristics of distinct ointments vary depending on the kind and concentration of additives (macrogol). Zhang et al. in [21] analyzed degree-based topological indexes and regression models for nine anti-malaria medicines. Regression models are used to analyze the physicochemical features of six anti-malaria medicines. After analyzing the results, conclusions were generated based on numerous statistical factors. QSPR study has been conducted on the characteristics and topological indices of Alzheimer disease medication structures by Ashraf et al. in [22]. The study found a link between particular topological indices and therapeutic efficacy, shedding light on key structural elements for effective Alzheimer’s treatment.

This approach shows promise for rational medication design and optimization in the search for new Alzheimer’s therapies. This study used Multi-Criteria Decision-Making (MCDM) techniques, such as TOPSIS and SAW, to examine how the chemical structure of potential Alzheimer disease (AD) drugs affects their therapeutic efficacy. In [23] Hui et al. used linear and multiple regression models to estimate the physical and chemical properties of antiemetic drugs by using topological descriptors as independent variables. Moreover, a Maple-based algorithm was developed to compute the degree-based topological descriptors. Fan et al. [24] created MAPLE-based code to calculate the reducible ve-degree topological descriptors of NSAIDs. A linear regression model was used to predict four physicochemical features of 70 NSAIDs. Two physical variables, Molecular Weight and Complexity, have a high association with reducible degree-based topological descriptors. In both cases, the correlation coefficient is greater than 0.90. We created quadratic and cubic regression models and compared their results. These findings may improve our understanding of NSAID drug structures and predict their pharmacological efficacy. The focus of mathematical chemistry is on topological indices, which are used in quantitative structure property relationship (QSPR) models to predict the properties of chemical compounds in order to save time and money. The COVID-19 pandemic is often regarded as the most severe crisis in modern medicine. Researchers have evaluated a number of antiviral medications that are available to treat COVID-19, and some have discovered that they aid in the removal of this virus. The study in [25] created and analyzed curvilinear and multilinear regression models to predict the physicochemical features of antiviral medicines using specified indices. The models and results of this investigation will help identify new medications to treat COVID-19. Parveen et al. [26,27] computed *TIs* and connected them to the linear QSPR model for medications used to treat rheumatoid arthritis (RA) and diabetes. The pharmaceutical manufacturing sectors will use the findings to help develop new medications and preventative strategies for a variety of diseases. The range of *TIs* for medications is significantly influenced by the correlation coefficient. These findings open the eyes of pharmaceutical researchers that study medication science and offer a way to forecast and estimate the physicochemical characteristics of novel RA treatment medications that are intended to treat other specific autoimmune disorders.

In [28] degree-based topological parameters for medications used to treat the infertility were computed and used quadratic, cubic, exponential, and logarithmic regression models to perform a QSPR analysis on the anticipated degree-based topological descriptors. In [29] QSPR analysis of some degree based topological indices is examined, and it is demonstrated that there is a strong correlation between these indices and the physical characteristics of anti-TB medications. This analysis could aid chemists and pharmaceutical business professionals in forecasting the characteristics of anti tuberculosis medications without doing experiments. Synthetic antibiotics called quinolones are used to treat bacterial infections. Several Quinolone antibiotic medications are studied by using QSPR analysis to create topological indices [30]. For every topological index, curvilinear regression models, including linear, quadratic, and cubic regression models are identified. Ravi and Desikan calculated the reduced reverse degree based indices for a family of benzenoid hydrocarbon to perform QSPR analysis [31]. They demonstrated how well the reduced reverse degree based indices can forecast every property taken into account for the benzenoid hydrocarbons. Additionally, they compared the 16 degree-based indiced considered with the prediction power of reduced reverse degree-based topological descriptors. The porphyrazine structure, noted for its excellent chemical and thermal stability, has gained attention in materials science, chemical re activity, functionalization and drug creation. Entropy measure aids in assessing the material’s stability and behavior in various situations. Furthermore, a greater comprehension of the intricate characteristics of porphyrazine is made possible by the use of logarithmic regression models to establish correlations between these indices and entropy by Khalid et al. in [32]. They calculated the entropy of the porphyrazine structure using the M-polynomial to obtain molecular descriptors for degree-based topological indices. A computational approach to design drug for multiple diseases via QSPR modeling and multi-criteria decision analysis is the new and significant area of research in chemical graph theory [33,34]. Following the applications of topological indices in forecasting the physicochemical properties this article presents the correlation between amino acids and Topological indices. In this article 13 amino acids and 11 properties are under consideration. First we calulated the topological indices (family of Gourava indices) i.e first and second Gourva index, first and second hyper Gourava index, first and second Alpha Gourava index, first and second Gamma Gourava index of 13 amino acids. Then we performed the curvilinear regression and multiple linear regression and the analyzed the results.

## Significance of this work

Family of Gourava indices are computed for amino acids and their physicochemical properties are used to do QSPR analysis.

- Amino acids are taken into consideration, in this study, to fit the curvilinear regression models.
- While most research use linear regression models, this study is different in that it extends the regression models for cubic and quadratic, logarithmic, exponential and multiple forms for improved efficacy.
- In order to analyze the physicochemical characteristics of the medications under consideration, a number of statistical parameters are calculated and compared with the topological indices.
- Graphical comparison of regression models is shown.

## Materials and methods

**Fig 1:**
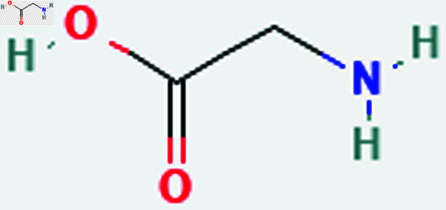
Glycine (Gly).

**Fig 2:**
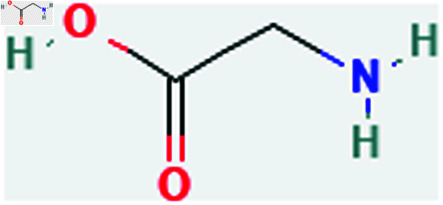
Alanine (Ala).

**Fig 3:**
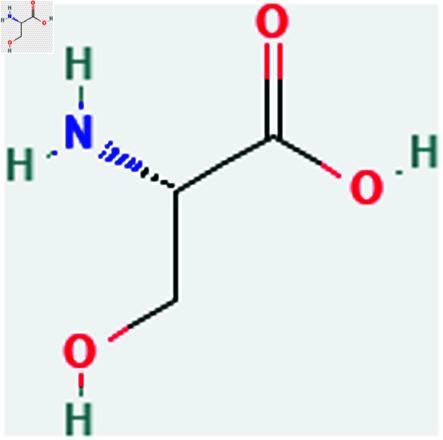
Serine (ser).

**Fig 4:**
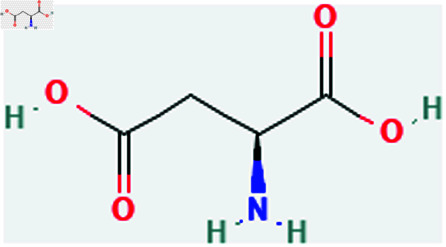
Aspartic acid.

**Fig 5:**
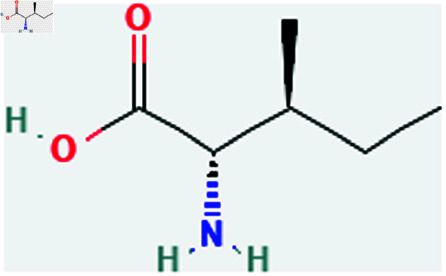
Isolucine (ile).

**Fig 6:**
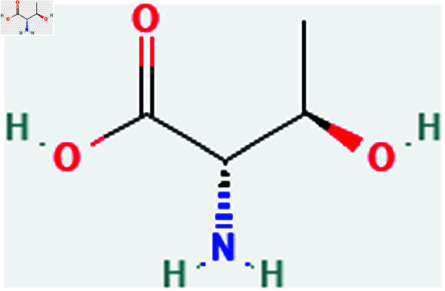
Threonine (Thr).

**Fig 7:**
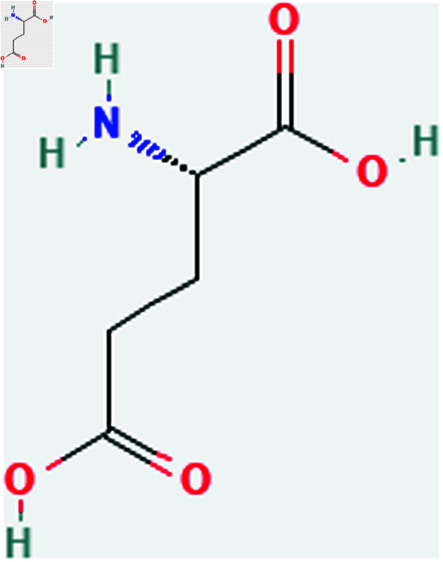
Glutamic acid (Glu).

**Fig 8:**
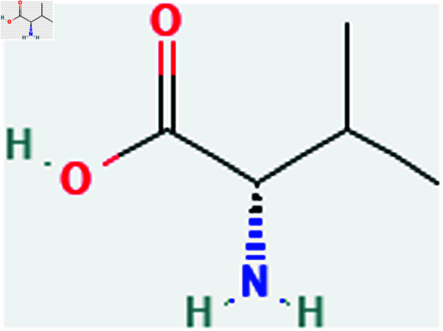
Valline (Val).

**Fig 9:**
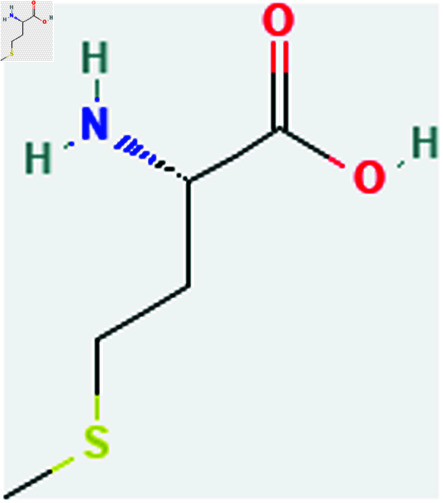
Methionine (Met).

**Fig 10:**
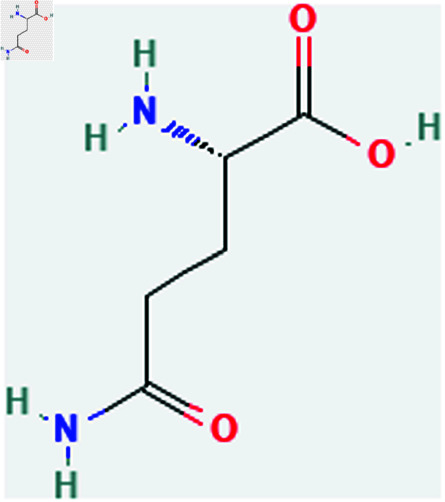
Glutamine (Gin).

**Fig 11:**
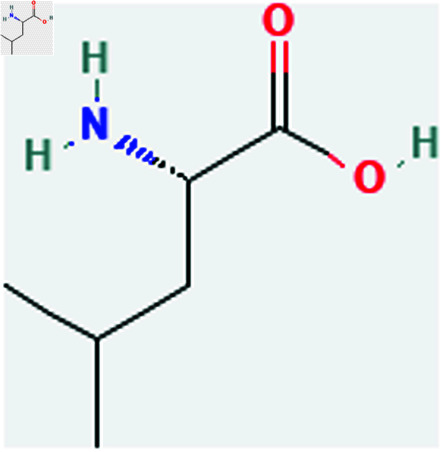
Leucine (Leu).

**Fig 12:**
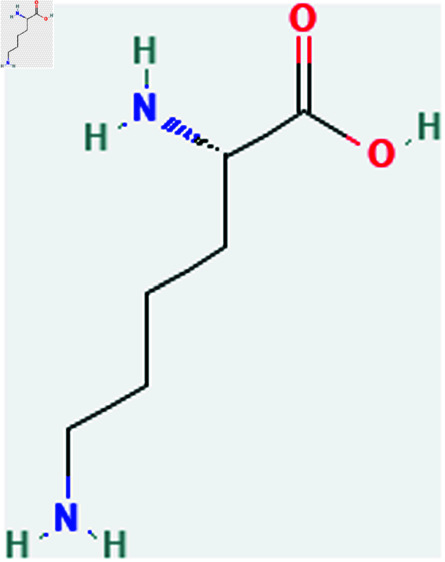
Lysine (Lys).

**Fig 13:**
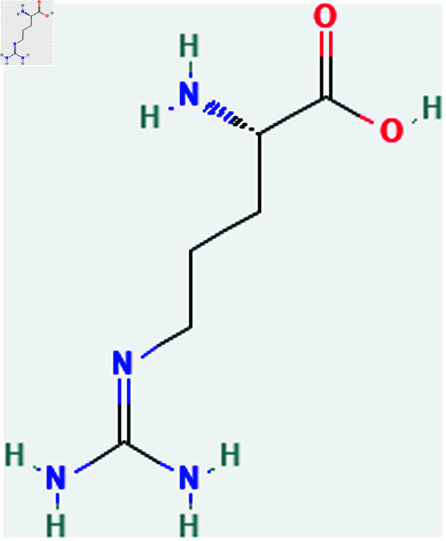
Arginie (arg).

Only connected, simple, undirected graphs are considered in this study. Assume *G* is exactly a graph. Then *V*(*G*) and *E*(*G*) signify its vertex and edge sets, respectively. Here $d_{G}(\mu )$ represent the degree of a vertex but we use *d*(*G*) rather than $d_{G}(\mu )$ when there is no ambiguity about the graph under account. In this article we are considering 13 amino acids present in Figs 1-13. Pharmaceutical industry, the physicochemical characteristics of amino acids are extremely important since they have a direct impact on therapeutic agent bioavailability, drug delivery, and drug design. Properties of these amino acids are presented in Table 1 Some of these characteristics have the following effects on pharmaceutical science.

**Table 1:** Properties of amino acids.

Name of Amino Acids	Da	MP	Pl	pKa1	pKa2	HI	*A* ^3^	LogP	D	FP	pH
*Glycine*(*Gly*)	75.07	233	5.97	2.34	9.6	-0.4	60.1	-1.39	1.1607	7	0
*Alanine*(*Ala*)	89.09	258	6.01	2.34	9.69	1.8	88.6	-2.83	1.424	7.5	41
*Serine*(*Ser*)	105.09	246	5.68	2.21	9.15	-0.8	89	-1.75	1.603	7.5	-5
*Asparticacid*(*asp*)	133.1	270	2.77	1.88	9.6	-3.5	111.1	-1.67	1.7	5	-55
*Isolucine*(*ile*)	131.18	285.5	6.02	2.36	9.68	4.5	166.7	0.41	1.207	5.5	99
*Threonine*(*Thr*)	119.12	265	5.6	2.09	9.1	-0.7	116.1	-1.43	1.3	6	13
*Glutamicacid*(*Glu*)	147.13	199	3.22	2.19	9.67	-3.5	138.4	-1.39	1.4601	6.5	-31
*Valline*(*Val*)	117.15	298	5.96	2.32	9.62	4.2	140	-0.01	1.316	6.5	76
*Methionine*(*Met*)	149.21	281	5.74	2.28	9.21	1.9	162.9	-0.56	1.34	2.5	74
*Glutamine*(*Gin*)	146.15	185	5.65	2.17	9.13	-3.5	143.8	-2.05	1.47	4	-10
*Leucine*(*Leu*)	131.18	293	5.98	2.36	9.6	3.8	166.7	-1.62	1.17	9	97
*Lysine*(*Lys*)	146.19	224.5	9.74	2.18	8.95	-3.9	168.6	-1.15	1.1	6	-23
*Arginine*(*Arg*)	174.2	260	10.76	2.17	9.04	-4.5	173.4	-1.43	1.5	5	-14

**Molecular Weight (Da)** affects the drug’s capacity to pass through cell membranes. Drugs based on lower molecular weight amino acids frequently have improved distribution and absorption in the body. **Melting Point (MP) ** affects the stability of drugs while they are being processed and stored. **Isoelectric Point (Pl)** establishes the neutral pH of an amino acid-based medication, affecting its solubility, stability, and physiological pH interactions. **pKa values** are the values that alter a drug’s solubility and permeability by influencing its ionization at physiological pH. Understanding the pKa is essential for forecasting a drug’s behavior in different bodily compartments and its possible ability to attach to enzymes or receptors. **Hydropathy Index (HI)** is the ability to reflect hydrophilicity or hydrophobicity is crucial for medication formulation. **Side chain Volume (*A*^3^)** fits into active areas and influences the capacity to bind to particular receptors or enzymes.

**LogP** shows lipophilicity and affects distribution and absorption. **Density (D)** useful in protein crystallization and structural analysis. **Frequency of proteins (FP)** aids in the development of protein-based medications or bioinformatics tools for comprehending the functions and sequencing of proteins. **pH** controls their chemical interactions, behavior, and operation in pharmacological and biological systems.

Computations of topological indices i.e first and second Gourva index, first and second hyper Gourava index, first and second Alpha Gourava index, first and second Gamma Gourava index for these amino acids are presented in Table 2.

**Table 2:** Topological indices computed.

Name of Amino Acids	$GO_{1}$	$GO_{2}$	$HGO_{1}$	$HGO_{2}$	$AGO_{1}$	$AGO_{2}$	$GGO_{1}$	$GGO_{2}$
*Glycine*(*Gly*)	28	93	1177	16280	181	914	489	3184
*Alanine*(*Ala*)	135	448	1915	3364	271	1562	771	5752
*Serine*(*Ser*)	145	462	2055	35404	285	1664	793	6086
*Asparticacid*(*asp*)	168	560	2390	41200	326	1912	888	6896
*Isolucine*(*ile*)	181	602	2567	43468	351	1438	781	7322
*Threonine*(*Thr*)	187	650	2793	52588	375	2312	1075	8654
*Glutamicacid*(*Glu*)	199	728	3128	58384	416	2560	1170	9464
*Valline*(*Val*)	219	784	3391	67832	451	2858	1335	10888
*Methionine*(*Met*)	223	752	3207	56056	459	2666	1351	9992
*Glutamine*(*Gin*)	223	770	3305	60654	441	2586	1063	9890
*Leucine*(*Leu*)	261	952	4129	85016	541	3500	1617	13456
*Lysine*(*Lys*)	285	1040	4507	91672	886	3798	1713	14456
*Arginine*(*Arg*)	296	1082	4414	88978	584	3592	1640	13172

## Regression models to predict physicochemical properties

Regression is a statistical technique that models and examines the relationship between a dependent variable (such as the physicochemical or biological properties of molecules) and one or more independent variables (such as molecular descriptors). Researchers can develop predictive models that aid in their comprehension of how modifications to molecular structure can impact different molecule behaviors and properties by using regression analysis. There are many types of regression. In this manuscript we are considering linear regression model, quadratic regression model, cubic regression model logarithmic regression model and multiple linear model. **Linear** regression model investigates how one independent variable (*X*) and one dependent variable (*Y*) relate to one another. **Quadratic** allows for one curve and depicts a parabolic relationship between *X* and *Y*. **Cubic** allows for two inflection points or a S-shaped curve, capturing more intricate relationships. **Logarithmic** regression demonstrates relationships in which dwindling returns occur when *X* increases and changes in *Y* decrease. **Multiple** linear simulates how two or more independent variables relate to one dependent variable. Equation for the proposed models are as follows:

$Y=a+\beta_{1}X$ (linear)
$Y=a+\beta_{1}X+\beta_{2}X^{2}$ (quadratic)
$Y=a+\beta_{1}X+\beta_{2}X^{2}+\beta_{3}X^{3}$ (cubic)
*Y* = *a* + *ln* ( *X* )  (logarithmic)
$Y=a+\beta_{1}X_{1}+\beta_{2}X_{2}+\beta_{3}X_{3}+...+\beta_{n}X_{n}$ (multiple)

Where ***a*** is the intercept value of *Y* when *X* = 0. **$\beta_{i}$** where  ( *i* = 1 , 2 , 3 . . . *n* )  is the slope for a unit change. It evaluates how each independent variable affects the dependent one. With all other variables held constant, it displays the change in *Y* for a one-unit change in *X*. ***R*** or (r) is the correlation coefficient it determines whether two variables are related to one another. It ranges form 0 to 1. *R* = 1 shows perfect positive correlation and *r* = − 1 shows perfect negative correlation and *R* = 0 means no correlation. ***R*^2^** is the coefficient of determination. It represents the proportion of variance in the dependent variable explained by the independent variable(s). It ranges from 0 to 1, higher values indicate better fit. **Adj(*R*^2^**) is to adjust *R*^2^ to avoid the over fitting in the model. **Standard Error of Estimate (SEE)** calculates how far away the observed values are on average from the regression line. Better model accuracy is indicated by lower numbers. A **p-value** indicates the significance of the model. If p-value <0.05 the model is statistically significant. **F-value** represents the general significance of the regression model. In this work Physical properties of amino acids are considered as dependent factors and Gourava indices are considered as independent variables.

### Calculation of statistical parameters

This section provides the statistical analysis of regression models considered in this work using SPSS software. Results of linear, quadratic, cubic, logarithmic and exponential regression are presented in Tables 3-10.

**Table 3:** Statical parameters for QSPR model for *GO*_1_.

Properties	a	$\beta_{1}$	$\beta_{2}$	$\beta_{3}$	R	*R* ^2^	Adj *R*^2^	SEE	F	P
**Linear regression**										
Da	48.840	0.392	–	–	0.860	0.74	0.716	14.345	31.246	<0.001
Pl	1.950	0.020	–	–	0.564	0.318	0.256	1.849	5.129	0.045
*A* ^3^	19.492	0.561	–	–	0.900	0.810	0.793	16.749	46.912	<0.001
**Quadratic regression**										
Da	0.216	0.922	-0.001	–	0.880	0.775	0.730	13.978	17.248	<0.001
pl	15.018	-0.122	0.000	–	0.851	0.724	0.668	1.235	13.095	0.002
*A* ^3^	-55.201	1.375	-0.002	–	0.925	0.855	0.826	15.345	29.498	<0.001
**Cubic regression**										
Da	-95.8081	2.630	-0.011	1.587E-5	0.888	0.789	0.719	14.269	11.234	0.002
Pl	1.203	0.124	-0.001	2.284E-6	0.877	0.769	0.692	1.189	10.001	0.003
*A* ^3^	-10.556	0.581	0.002	-7.381E-6	0.926	0.857	0.809	16.084	17.932	<0.001
**Logarithmic regression**										
Da	73.317	-257.915	–	–	0.882	0.778	0.757	13.257	38.466	<0.001
Pl	-9.517	2.964	–	–	0.448	0.200	0.128	2.003	2.755	0.125
*A* ^3^	-418.173	104.663	–		0.921	0.848	0.834	14.980	61.391	<0.001
**Exponential regression**										
Da	63.681	0.003	–	–	0.857	0.735	0.710	0.124	30.432	<0.001
Pl	3.245	0.003	–	–	0.465	0.216	0.145	0.332	3.033	0.109
*A* ^3^	47.247	0.005	–	–	0.893	0.797	0.778	0.153	43.094	<0.001

**Table 4:** Statical parameters for QSPR model for *GO*_2_.

Properties	a	$\beta_{1}$	$\beta_{2}$	$\beta_{3}$	R	*R* ^2^	Adj *R*^2^	SEE	F	P
**Linear regression**										
Da	72.229	0.82	–	–	0.851	0.724	0.698	14.780	28.799	<0.001
Pl	3.510	0.004	–	–	0.493	0.243	0.174	1.948	3.535	0.087
*A* ^3^	52.740	0.117	–	–	0.893	0.797	0.778	17.325	43.122	<0.001
**Quadratic regression**										
Da	69.329	0.095	-1.085E-5	–	0.852	0.725	0.671	15.449	13.212	.002
pl	6.899	1.268E-5	-0.011	–	0.800	0.641	0.569	1.408	8.908	0.006
*A* ^3^	-55.201	1.375	-0.002	–	0.893	0.798	0.757	18,127	19.721	<0.001
**Cubic regression**										
Da	71.705	0.051	8.998E-5	-5.934E-8	0.853	0.728	0.638	16.199	8.043	0.006
Pl	5.69	3.011E-8	-3.848E-5	3.011E-8	0.871	0.758	0.677	1.218	9.386	0.004
*A* ^3^	60.497	-2.662E-7	0.000	-0.066	0.910	0.829	0.772	17.576	14.530	<0.001
**Logarithmic regression**										
Da	-8.018	21.537	–	–	0.750	0.563	0.523	18.580	14.182	0.003
Pl	3.324	0.437	–	–	0.191	0.037	-0.051	2.198	0.417	0.531
*A* ^3^	-58.325	30.239	–	–	0.771	0.594	0.557	24.483	16.102	0.002
**Exponential regression**										
Da	76.952	0.001	–	–	0.866	0.750	0.727	0.120	32.962	<0.001
Pl	4.058	0.001	–	–	0.397	0.158	0.081	0.344	2.061	0. 179
*A* ^3^	61.934	0.001	–	–	0.910	0.829	0.813	0.140	53.218	<0.001

**Table 5:** Statical parameters for QSPR model for *HGO*_1_.

Properties	a	$\beta_{1}$	$\beta_{2}$	$\beta_{3}$	R	*R* ^2^	Adj *R*^2^	SEE	F	P
**Linear regression**										
Da	61.633	0.022	–	–	0.817	0.667	0.637	16.214	22.072	0.001
Pl	2.489	0.001	–	–	0.556	0.309	0.246	1.862	4.913	0.049
*A* ^3^	34.863	0.033	–	–	0.881	0.777	0.756	18.156	38.281	0.000
**Quadratic regression**										
Da	20.952	-6.404E-6	0.60	–	0.855	0.731	0.677	15.307	13.553	0.001
pl	12.025	-0.006	1.205E-6	–	0.813	0.661	0.593	1.368	9.745	0.004
*A* ^3^	-25.815	1.078	-7.667E-6	–	0.908	0.825	0.790	16.851	23.606	0.000
**Cubic regression**										
Da	-21.121	0.101	-2.196E-5	1.806E-9	0.857	0.734	0.645	16.029	8.279	0.006
Pl	3.234	0.005	-3.059E-6	4.950E-10	0.838	0.702	0.603	1.351	7.082	0.010
*A* ^3^	-13.012	0.062	-1.457E-6	-7.208E-10	0.909	0.826	0.767	17.747	14.193	0.001
**Logarithmic regression**										
Da	-355.370	60.826	–	–	0.851	0.724	0.699	14.760	28.907	0.000
Pl	-13.299	2.439	–	–	0.428	0.184	0.109	2.024	2.473	0.144
*A* ^3^	-569.839	88.411	–		0.905	0.819	0.802	16.352	49.756	<0.000
**Exponential regression**										
Da	70.753	0.000	–	–	0.820	0.672	0.642	0.138	22.511	0.001
Pl	3.76	0.000	–	–	0.463	0.215	0.143	0.333	3.006	0.111
*A* ^3^	53.937	0.000	–	–	0.876	0.767	0.746	0.163	36.271	0.000

**Table 6:** Statical parameters for QSPR model for *HGO*_2_.

Properties	a	$\beta_{1}$	$\beta_{2}$	$\beta_{3}$	R	*R* ^2^	Adj *R*^2^	SEE	F	P
**Linear regression**										
Da	84.690	0.001	–	–	0.793	0.629	0.595	17.133	18.616	0.001
Pl	3.924	4.007E-5	–	–	0.497	0.247	0.178	1.944	3.601	0.084
*A* ^3^	70.246	0.001	–	–	0.837	0.700	0.673	21.037	25.710	0.000
**Quadratic regression**										
Da	72.789	0.001	-6.395E-9	–	0.814	0.663	0.595	15.449	17.126	0.004
pl	7.062	0.000	1.686E-9	–	0.787	0.619	0.543	1.449	8.140	0.008
*A* ^3^	-55.201	1.375	-0.002	–	0.839	0.705	0.646	21.904	11.929	0.002
**Cubic regression**										
Da	77.318	0.001	1.055E-8	-1.159E-13	0.816	0.667	0.556	17.944	6.000	0.016
Pl	5.881	4.000E-5	-2.735E-9	3.022E-14	0.814	0.633	0.550	1.438	5.896	0.017
*A* ^3^	80.494	-0.001	5.688E-8	-4.101E-13	0.855	0.732	0.642	22.006	8.183	0.006
**Logarithmic regression**										
Da	-112.000	22.495	–	–	0.746	0.557	0.517	18.707	13.842	0.003
Pl	0.127	0.558	–	–	0.233	0.054	-0.032	2.178	0.629	0.444
*A* ^3^	-186.205	29.895	–	–	0.726	0.527	0.484	26.441	12.237	0.005
**Exponential regression**										
Da	76.952	0.001	–	–	0.798	0.636	0.603	0.145	19.230	0.001
Pl	4.279	5.464E-6	–	–	0.404	0.163	0.087	0.343	2.146	0. 171
*A* ^3^	73.941	1.004E-5	–	–	0.823	0.678	0.648	0.192	23.135	0.001

**Table 7:** Statical parameters for QSPR model for *AGO*_1_.

Properties	a	$\beta_{1}$	$\beta_{2}$	$\beta_{3}$	R	*R* ^2^	Adj *R*^2^	SEE	F	P
**Linear regression**										
Da	83.924	0.103	–	–	0.793	0.628	0.0.594	17.140	18.591	0.001
Pl	2.794	0.008	–	–	0.633	0.400	0.346	1.734	7.342	0.020
*A* ^3^	64.715	0.159	–	–	0.762	0.581	0.542	24.890	15.222	0.002
**Quadratic regression**										
Da	2.518	0.459	0.000	–	0.865	0.748	0.697	14.805	14.831	0.001
pl	5.230	-0.003	1.001E-5	–	0.664	0.442	0.330	1.755	3.953	0.054
*A* ^3^	-40.665	0.619	0.000	–	0.918	0.843	0.811	15.982	26.803	0.000
**Cubic regression**										
Da	-5.678	0.519	0.000	8.331E-8	0.865	0.748	0.664	15.593	8.918	0.005
Pl	18.930	-0.104	0.000	-1.393E-7	0.790	0.624	0.498	1.519	4.969	0.026
*A* ^3^	80.494	-0.001	5.688E-8	-4.101E-13	0.918	0.843	0.791	16.838	16.099	0.001
**Logarithmic regression**										
Da	-195.661	54.054	–	–	0.793	0.628	0.594	17.140	18.591	0.001
Pl	-11.219	2.890	–	–	0.532	0.0.283	0.218	1.896	4.342	0.061
*A* ^3^	-351.424	80.859	–	–	0.867	0.752	0.730	19.125	33.417	0.000
**Exponential regression**										
Da	85.781	0.001	–	–	0.675	0.456	0.406	0.178	9.203	0.011
Pl	3.596	0.001	–	–	0.537	0.289	0.224	0.317	4.464	0.058
*A* ^3^	70.922	0.001	–	–	0.742	0.550	0.509	0.227	13.438	0.004

**Table 8:** Statical parameters for QSPR model for *AGO*_2_.

Properties	a	$\beta_{1}$	$\beta_{2}$	$\beta_{3}$	R	*R* ^2^	Adj *R*^2^	SEE	F	P
**Linear regression**										
Da	73.798	0.022	–	–	0.744	0.554	0.514	18.772	13.671	0.004
Pl	3.019	0.001	–	–	0.529	0.279	0.214	1.901	4.267	0.063
*A* ^3^	55.213	0.032	–	–	0.779	0.607	0.571	24.102	16.965	0.002
**Quadratic regression**										
Da	30.461	0.063	-8.417E-6	–	0.781	0.610	0.532	18.407	7.830	0.009
pl	11.631	-0.007	1.673E-6	–	0.793	0.629	0.555	1.431	8.474	0.007
*A* ^3^	31.808	0.054	-4.546E-6	–	0.784	0.615	0.538	24.996	8.001	0.008
**Cubic regression**										
Da	-13.997	0.132	-4.018E-5	4.457E-9	0.787	0.619	0.492	19.184	4.875	0.028
Pl	6.654	0.001	-1.883E-6	4.989E-10	0.804	0.646	0.528	1.473	5.479	0.020
*A* ^3^	-14.818	-1.26	-3.786E-8	4.674E-9	0.788	0.621	0.494	26.171	4.906	0.027
y**Logarithmic regression**										
Da	-262.125	50.562	–	–	0.781	0.610	0.574	17.563	17.185	0.002
Pl	-10.080	2.095	–	–	0.406	0.165	-0.089	2.046	2.173	0.168
*A* ^3^	-405.101	69.706	–	–	0.787	0.620	0.586	23.685	17.959	0.001
**Exponential regression**										
Da	78.648	0.000	–	–	0.745	0.555	0.514	0.161	13.696	0.003
Pl	3.760	0.000	–	–	0.436	0.190	0.117	0.338	2.585	0. 136
*A* ^3^	64.223	0.000	–	–	0.778	0.606	0.570	0.213	16.901	0.002

**Table 9:** Statical parameters for QSPR model for *GGO*_1_.

Properties	a	$\beta_{1}$	$\beta_{2}$	$\beta_{3}$	R	*R* ^2^	Adj *R*^2^	SEE	F	P
**Linear regression**										
Da	69.624	0.052	–	–	0.737	0.544	0.0.502	18.990	13.107	0.004
Pl	2.662	0.003	–	–	0.543	0.295	0.231	1.881	4.595	0.055
*A* ^3^	45.153	0.078	–	–	0.809	0.655	0.624	22.573	20.883	0.001
**Quadratic regression**										
Da	3.223	0.181	-5.628E-5	–	0.789	0.623	0.547	18.111	8.253	0.008
pl	13.070	-0.017	8.823E-6	–	0.775	0.601	0.521	1.484	7.520	0.010
*A* ^3^	-7.875	0.181	-4.495E-5	–	0.826	0.682	0.618	22.731	10.721	0.003
**Cubic regression**										
Da	-76.992	0.438	0.000	7.619E-8	0.799	0.639	0.518	18.683	5.303	0.022
Pl	4.426	0.010	-1.831E-5	8.211E-9	0.794	0.630	0.506	1.506	5.506	0.025
*A* ^3^	-96.510	0.465	0.000	8.419E-8	0.832	0.692	0.590	23.565	6.752	0.011
**Logarithmic regression**										
Da	-268.991	56.949	–	–	0.780	0.608	0.572	17.603	17.055	0.002
Pl	-11.813	2.567	–	–	0.441	0.195	0.122	1.896	2.660	0.131
*A* ^3^	-444.953	82.870	–	–	0.830	0.689	0.661	21.435	24.357	0.000
**Exponential regression**										
Da	75.885	0.000	–	–	0.738	0.544	0.503	0.162	13.133	0.004
Pl	3.544	0.000	–	–	0.457	0.209	0.137	0.334	2.906	0.116
*A* ^3^	59.192	0.001	–	–	0.801	0.642	0.610	0.203	19.740	0.001

**Table 10:** Statical parameters for QSPR model for *GGO*_2_.

Properties	a	$\beta_{1}$	$\beta_{2}$	$\beta_{3}$	R	*R* ^2^	Adj *R*^2^	SEE	F	P
**Linear regression**										
Da	71.228	0.006	–	–	0.764	0.584	0.546	18.140	15.420	0.002
Pl	2.989	0.000	–	–	0.538	0.290	0.225	1.887	4.492	0.058
*A* ^3^	45.455	0.010	–	–	0.859	0.738	0.715	19.660	30.032	0.000
**Quadratic regression**										
Da	15.568	0.020	-7.570E-7	–	0.829	0.688	0.626	16.468	11.029	0.003
pl	9.841	-0.001	9.444E-8	–	0.739	0.548	0.455	1.583	6.014	0.019
*A* ^3^	-9.197	0.023	-7.297E-7	–	0.889	0.790	0.748	18.461	18.834	0.000
**Cubic regression**										
Da	19.057	0.019	-5.679E-7	-7.156E-12	0.833	0.688	0.584	17.356	6.620	0.012
Pl	8.010	-0.001	-4.786E-9	3.755E-12	0.741	0.550	0.399	1.662	3.661	0.057
*A* ^3^	-12.707	0.025	-9.741E-7	9.249E-12	0.889	0.790	0.720	19.456	11.305	0.002
**Logarithmic regression**										
Da	-345.984	52.363	–	–	0.814	0.663	0.632	16.327	21.612	0.001
Pl	-13.093	2.119	–	–	0.413	0.171	0.096	2.039	2.268	0.16
*A* ^3^	-572.674	77.930	–	–	0.886	0.786	0.766	17.798	40.283	0.000
**Exponential regression**										
Da	76.534	5.357E-5	–	–	0.773	0.597	0.560	0.153	16.281	0.002
Pl	3.640	4.976E-5	–	–	0.460	0.212	0.140	0.333	2.953	0.114
*A* ^3^	59.127	8.344E-5	–	–	0.855	0.731	0.707	0.176	29.890	0.000

## Statistical analysis of regression models

### Linear regression models

Linear model of $GO_{1}$ shows a positive strong correlation with Da (R = 0.860), PI(0.564) and *A*^3^ (0.900). $GO_{2}$ shows a strong correlation with Da(0.851) and *A*^3^(0.893). $HGO_{1}$ shows a strong correlation with Da(0.817), *A*^3^(0.881) and PI(0.556). $HGO_{2}$ shows strong correlation with Da(0.793) and *A*^3^(0.837). $AGO_{1}$ shows a strong correlation with Da(0.793), PI(0.633) and *A*^3^(0.762). $AGO_{2}$ shows strong correlation with Da(0.744), PI(0.529) and *A*^3^(0.779). $GGO_{1}$ shows strong positive correlation with Da(0.737), PI(0.543) and *A*^3^(0.809). $GGO_{2}$ holds a positive strong correlation with Da(0.764), PI(0.538) and *A*^3^(0.859).

### Quadratic regression models

Quadratic model of $GO_{1}$ holds a positive strong correlation with Da (R=0.880), PI(0.851) and *A*^3^ (0.925). $GO_{2}$ shows a strong correlation with Da(0.852), PI(0.800) and *A*^3^(0.893). $HGO_{1}$ presents a strong correlation with Da(0.855), *A*^3^(0.908) and PI(0.813). $HGO_{2}$ shows strong correlation with Da(0.814), PI(0.787) and *A*^3^(0.839). $AGO_{1}$ shows a strong correlation with Da(0.865), PI(0.664) and *A*^3^(0.918). $AGO_{2}$ shows strong correlation with Da(0.781), PI(0.793) and *A*^3^(0.781). $GGO_{1}$ shows strong positive correlation with Da(0.789), PI(0.775) and *A*^3^(0.826).$GGO_{2}$ holds a positive strong correlation with Da(0.829), PI(0.739) and *A*^3^(0.889).

### Cubic regression models

Cubic model of $GO_{1}$ shows a positive strong correlation with Da (R=0.888), PI(0.877) and *A*^3^ (0.926). $GO_{2}$ shows a strong correlation with Da(0.853), PI(0.871) and *A*^3^(0.910). $HGO_{1}$ shows a strong correlation with Da(0.857), *A*^3^(0.838) and PI(0.909). $HGO_{2}$ shows strong correlation with Da(0.816), PI(0.814) and *A*^3^(0.855). $AGO_{1}$ shows a strong correlation with Da(0.865), PI(0.790) and *A*^3^(0.918). $AGO_{2}$ shows strong correlation with Da(0.787), PI(0.804) and *A*^3^(0.788). $GGO_{1}$ shows strong positive correlation with Da(0.799), PI(0.794) and *A*^3^(0.832). $GGO_{2}$ holds a positive strong correlation with Da(0.833), PI(0.741) and *A*^3^(0.889).

### Logarithmic regression models

Logarithmic model of $GO_{1}$ shows a positive strong correlation with Da (R=0.882) and *A*^3^ (0.921). $GO_{2}$ shows a strong correlation with Da(0.750) and *A*^3^(0.771). $HGO_{1}$ shows a strong correlation with Da(0.851) and *A*^3^(0.905). $HGO_{2}$ shows strong correlation with Da(0.746) and *A*^3^(0.726). $AGO_{1}$ shows a strong correlation with Da(0.793), PI(0.532) and *A*^3^(0.867). $AGO_{2}$ shows strong correlation with Da(0.781) and *A*^3^(0.787). $GGO_{1}$ shows strong positive correlation with Da(0.780) and *A*^3^(0.830). $GGO_{2}$ holds a positive strong correlation with Da(0.814) and *A*^3^(0.886).

### Exponential regresssion models

Logarithmic model of $GO_{1}$ shows a positive strong correlation with Da (R=0.857) and *A*^3^ (0.893). $GO_{2}$ shows a strong correlation with Da(0.866) and *A*^3^(0.910). $HGO_{1}$ shows a strong correlation with Da(0.820) and *A*^3^(0.876). $HGO_{2}$ shows strong correlation with Da(0.798) and *A*^3^(0.823). $AGO_{1}$ shows a positive correlation with Da(0.675), PI(0.537) and *A*^3^(0.742). $AGO_{2}$ shows strong correlation with Da(0.745) and *A*^3^(0.778). $GGO_{1}$ shows strong positive correlation with Da(0.738) and *A*^3^(0.801). $GGO_{2}$ holds a positive strong correlation with Da(0.773) and *A*^3^(0.855).

### Multiple linear regression models

When there are several predictors influencing the result, multiple regression works well. The regression statics for multiple linear model are presented in Table 11. Equation for multiple linear regression is as follows:


$$\begin{array}{ccc} Y=a+\beta_{1}GO_{1}+\beta_{2}GO_{2}+\beta_{3}HGO_{1}+\beta_{4}HGO_{2}+\beta_{5}AGO_{1}+ & & \\ \beta_{6}AGO_{2}+\beta_{7}GGO_{1}+\beta_{8}GGO_{2} \end{array}$$ (13)

$$\begin{array}{ccc} Da=38.289+2.317GO_{1}-0.160HGO_{1}+0.100GO_{2}+0.001HGO_{2}+ & & \\ 0.025AGO_{1}+0.018AGO_{2}-0.047GGO_{1}+0.000GGO_{2} \end{array}$$ (14)

$$\begin{array}{ccc} MP=278.964-3.141GO_{1}+0.223HGO_{1}+0.122GO_{2}+0.001HGO_{2}- & & \\ 1.58AGO_{1}-2.23AGO_{2}+0.526GGO_{1}-0.17GGO_{2} \end{array}$$ (15)

$$\begin{array}{ccc} PI=-3.104-0.095GO_{1}+0.025HGO_{1}-0.016GO_{2}- & & \\ 8.850E-5HGO_{2}+0.006AGO_{1}- & \\ 0.0038AGO_{2}+0.011GGO_{1}-0.004GGO_{2} \end{array}$$ (16)

$$\begin{array}{ccc} pKa1=1.748+0.002GO_{1}+0.000HGO_{1}-0.002GO_{2}-9.906E-6HGO_{2}- & & \\ 0.001AGO_{1}-0.001AGO_{2}+0.000GGO_{1}+0.000GGO_{2} \end{array}$$ (17)

$$\begin{array}{ccc} pKa2=9.744-0.051GO_{1}+0.006HGO_{1}-0.001GO_{2}-1.247E-5HGO_{2}- & & \\ 0.002AGO_{1}-0.001AGO_{2}+0.002GGO_{1}+0.000GGO_{2} \end{array}$$ (18)

$$\begin{array}{ccc} HI=0.510+0.017GO_{1}-0.014HGO_{1}-0.006GO_{2}-6.838E-5HGO_{2}- & & \\ 0.021AGO_{1}-0.019AGO_{2}+0.017GGO_{1}+0.009GGO_{2} \end{array}$$ (19)

$$\begin{array}{ccc} A^{3}=-5.067+2.404GO_{1}-1.85HGO_{1}+0.024GO_{2}+4.909E-5HGO_{2}- & & \\ 0.015AGO_{1}-0.094AGO_{2}+0.009GGO_{1}+0.045GGO_{2} \end{array}$$ (20)

$$\begin{array}{ccc} D=1.852+0.001GO_{1}+0.000HGO_{1}+0.002GO_{2}+5.643E-6HGO_{2}+ & & \\ 0.000AGO_{1}+0.001AGO_{2}+0.000GGO_{1}+0.000GGO_{2} \end{array}$$ (21)

$$\begin{array}{ccc} ph=-81.435+03.149GO_{1}-0.414HGO_{1}-0.200GO_{2}-0.002HGO_{2}- & & \\ 0.343AGO_{1}-0.299AGO_{2}+0.175GGO_{1}+0.175GGO_{2} \end{array}$$ (22)

$$\begin{array}{ccc} FoP=12.115-0.533GO_{1}+0.052HGO_{1}-0.006GO_{2}-3.686E-5HGO_{2}- & & \\ 0.008AGO_{1}-0.004AGO_{2}+0.015GGO_{1}-0.006GGO_{2} \end{array}$$ (23)

$$\begin{array}{ccc} logP=-1.360+0.033GO_{1}-0.005HGO_{1}+0.001GO_{2}+5.707E-5HGO_{2}+ & & \\ 0.001AGO_{1}-0.005AGO_{2}+0.006GGO_{1}+0.001GGO_{2} \end{array}$$ (24)

**Table 11:** Statics for multiple linear regression models.

Properties	R	*R* ^2^	Adj *R*^2^	SEE
Molar Weight (Da)	0.978	0.956	0.869	9.74873
Melting Point (MP)	0.862	0.743	0.230	30.64814
Isoelectric point (pI)	0.777	0.604	-1.87	2.33651
pka1 ( *α* − *COOH* )	0.865	0.749	2.46	0.11798
pka2 $\left(\alpha -NH_{3}\right)$	0.824	0.679	0.038	0.28249
Hydropathy Index (HI)	0.975	0.951	0.853	1.26880
Volume (*A*^3^)	0.993	0.986	0.959	7.49449
Density (D)	0.954	0.911	0.732	0.09371
Frequency of Proteins (FoP)	0.921	0.848	0.545	1.12703
pH	0.979	0.959	878	17.98898
logP	0.896	0.803	0.408	0.64953

### Graphical analysis

Graphical comparison of linear, quadratic, cubic, logarithmic and exponential regression is given in Figs 14-37 (properties of amino acids vs Topological indices). Graphs showing the results for multiple linear regression are presented in Figs 38-48.

**Fig 14:**
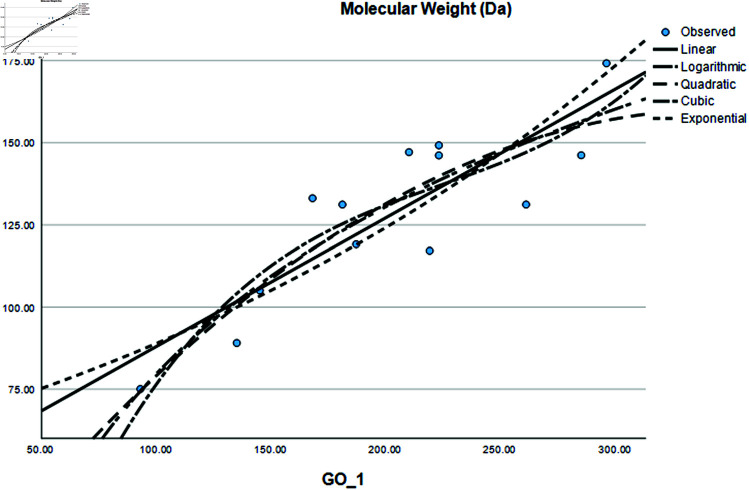
Comparison of linear, quadratic, cubic, logarithmic and exponential regression for Molecular Weight vs $GO_{1}$.

**Fig 15:**
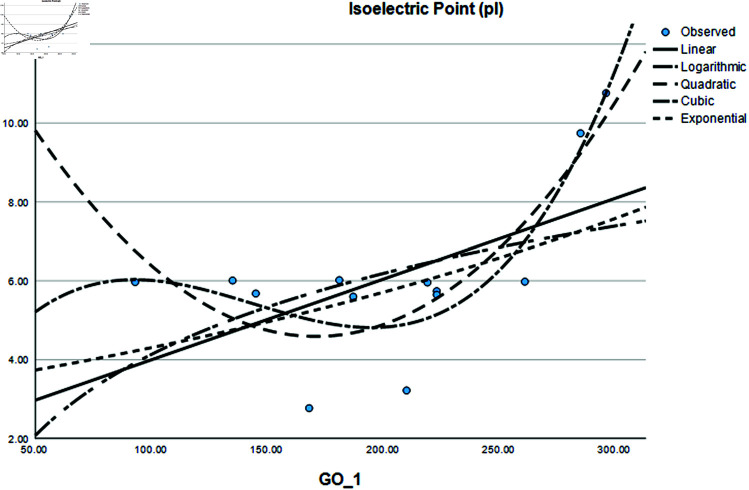
Comparison of linear, quadratic, cubic, logarithmic and exponential regression for Isoelectric Point (pl) vs $GO_{1}$.

**Fig 16:**
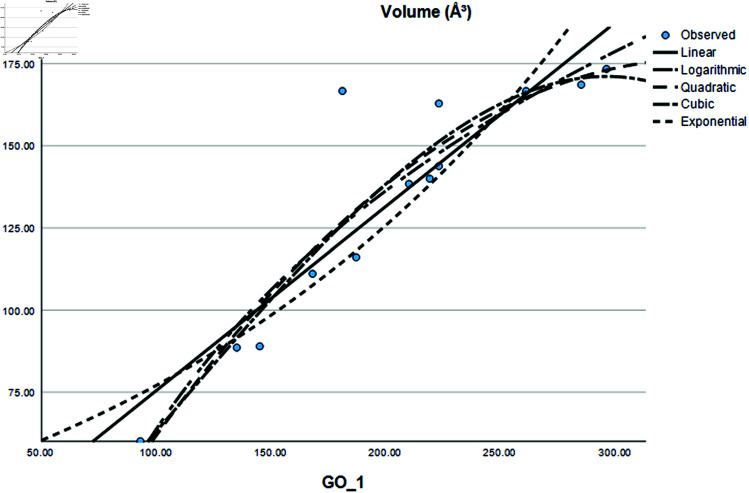
Comparison of linear, quadratic, cubic, logarithmic and exponential regression for Volume (*A*^3^) vs $GO_{1}$.

**Fig 17:**
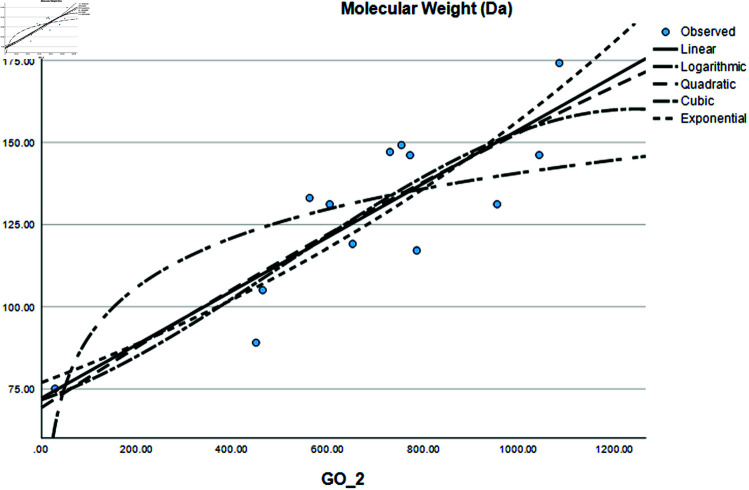
Comparison of linear, quadratic, cubic, logarithmic and exponential regression for Molecular Weight vs $GO_{2}$.

**Fig 18:**
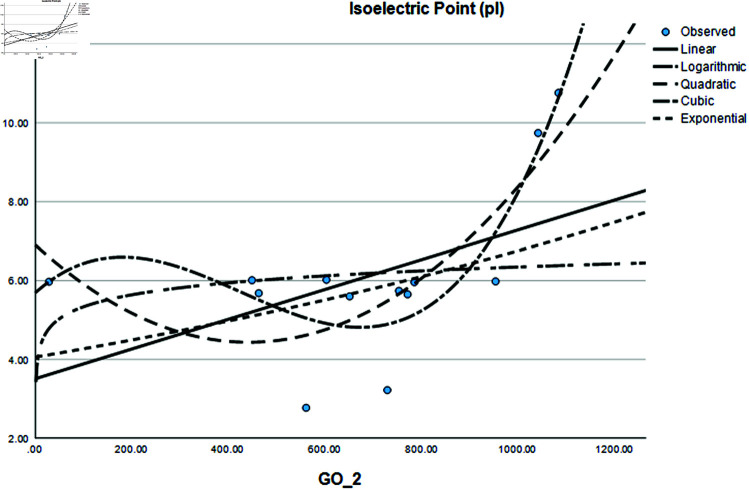
Comparison of linear, quadratic, cubic, logarithmic and exponential regression for Isoelectric Point (pl) vs $GO_{2}$.

**Fig 19:**
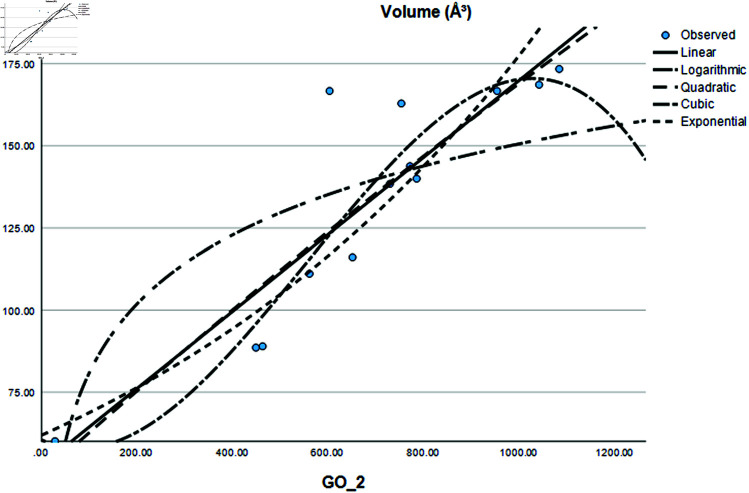
Comparison of linear, quadratic, cubic, logarithmic and exponential regression for Volume (*A*^3^) vs $GO_{2}$.

**Fig 20:**
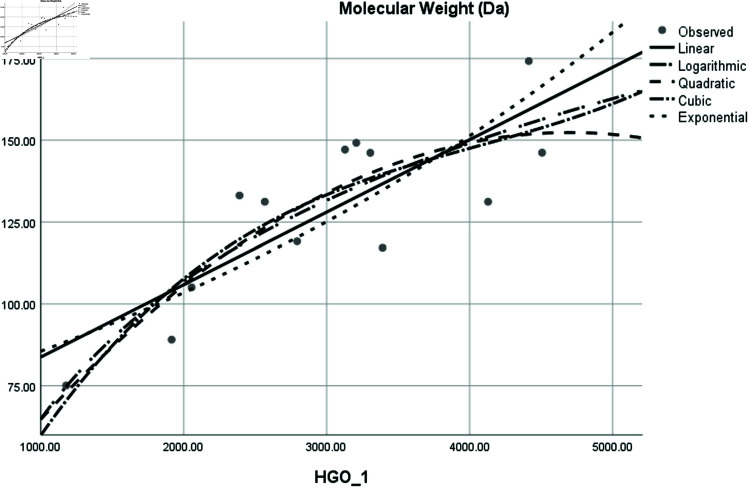
Comparison of linear, quadratic, cubic, logarithmic and exponential regression for Molecular Weight vs $HGO_{1}$.

**Fig 21:**
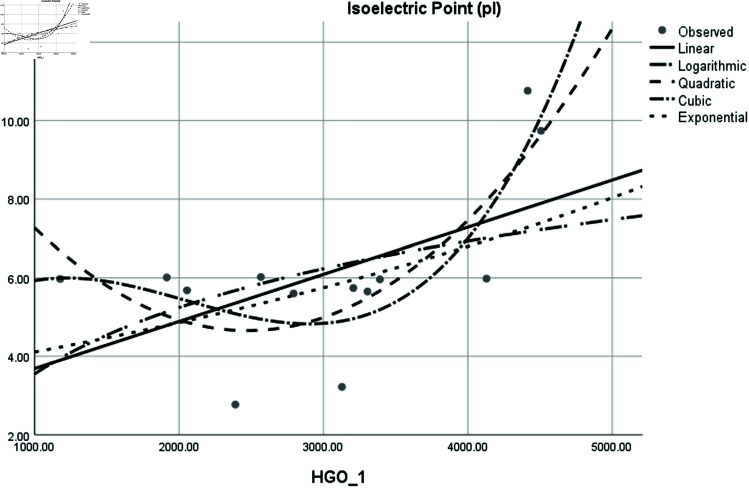
Comparison of linear, quadratic, cubic, logarithmic and exponential regression for Isoelectric Point (pl) vs $HGO_{1}$.

**Fig 22:**
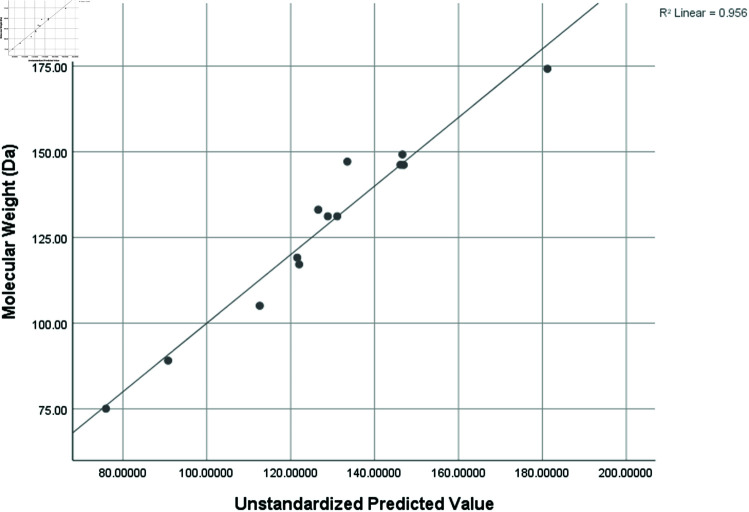
Comparison of linear, quadratic, cubic, logarithmic and exponential regression for Volume (*A*^3^) vs $HGO_{1}$.

**Fig 23:**
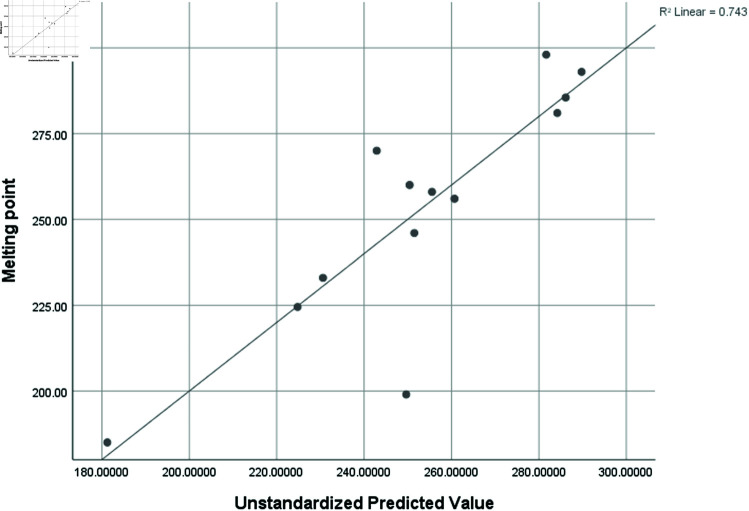
Comparison of linear, quadratic, cubic, logarithmic and exponential regression for Molecular Weight vs $HGO_{2}$.

**Fig 24:**
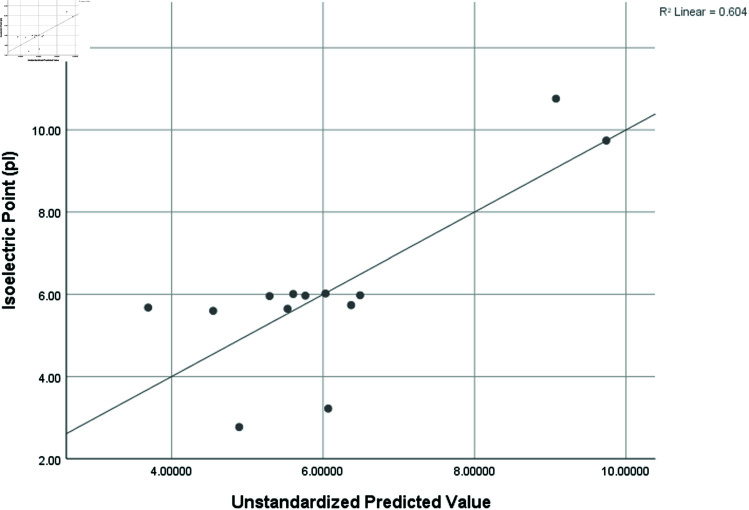
Comparison of linear, quadratic, cubic, logarithmic and exponential regression for Isoelectric Point (pl) vs $HGO_{2}$.

**Fig 25:**
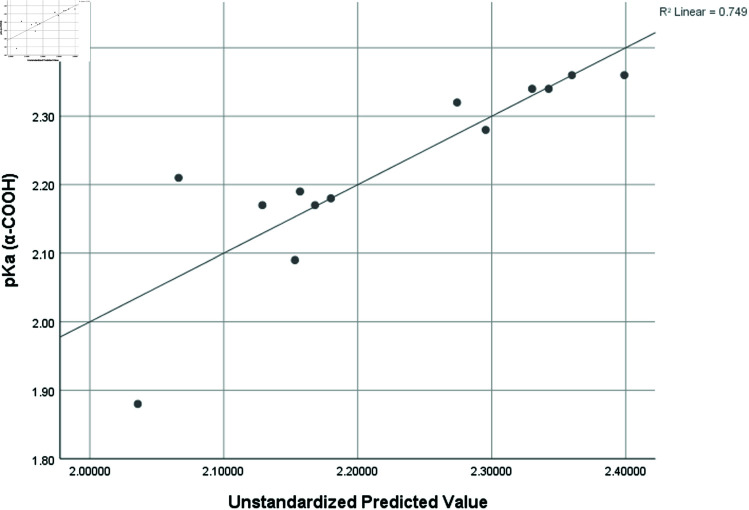
Comparison of linear, quadratic, cubic, logarithmic and exponential regression for Volume (*A*^3^) vs $HGO_{2}$.

**Fig 26:**
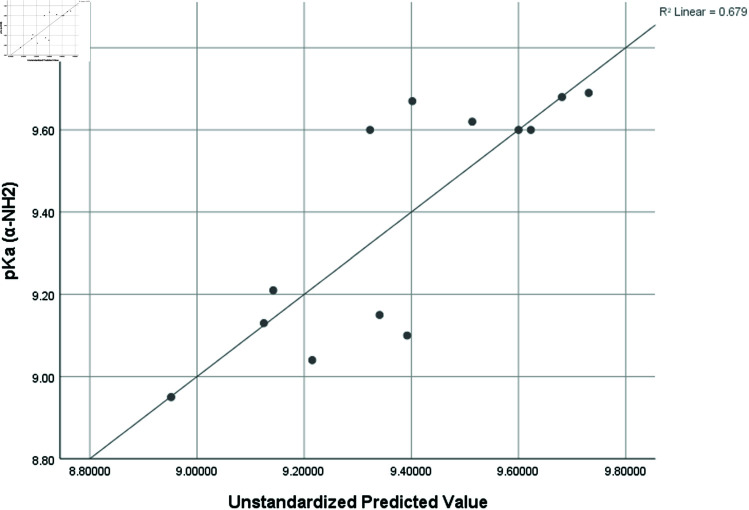
Comparison of linear, quadratic, cubic, logarithmic and exponential regression for Molecular Weight vs $AGO_{1}$.

**Fig 27:**
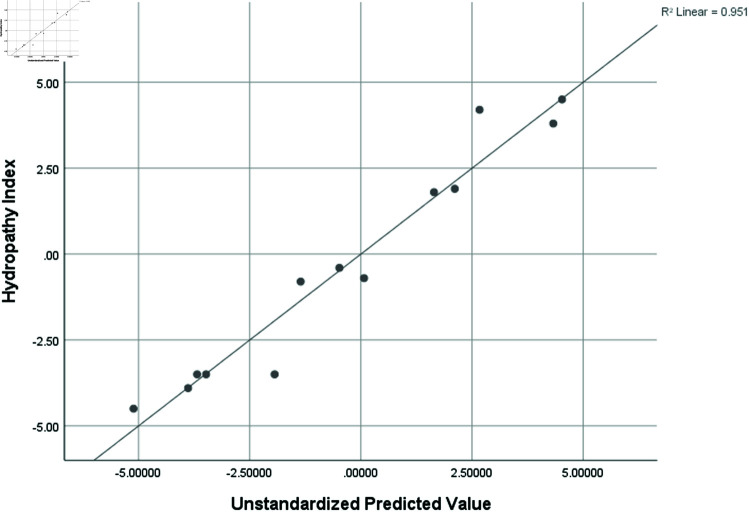
Comparison of linear, quadratic, cubic, logarithmic and exponential regression for Isoelectric Point (pl) vs $AGO_{1}$.

**Fig 28:**
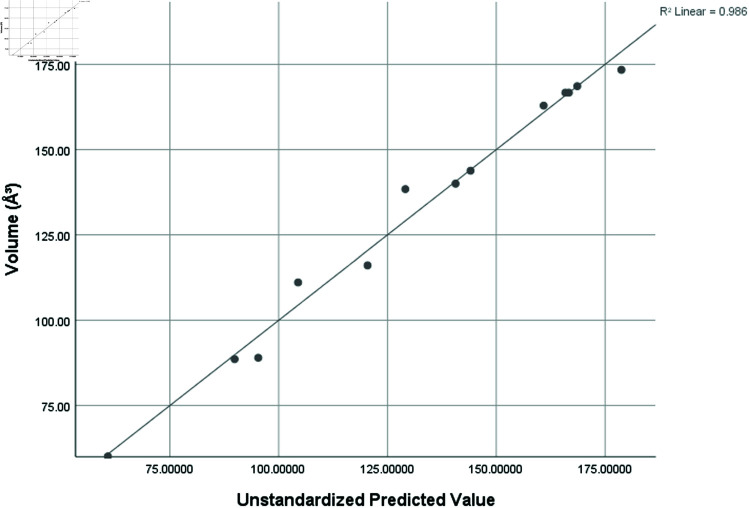
Comparison of linear, quadratic, cubic, logarithmic and exponential regression for Volume (*A*^3^) vs $AGO_{1}$.

**Fig 29:**
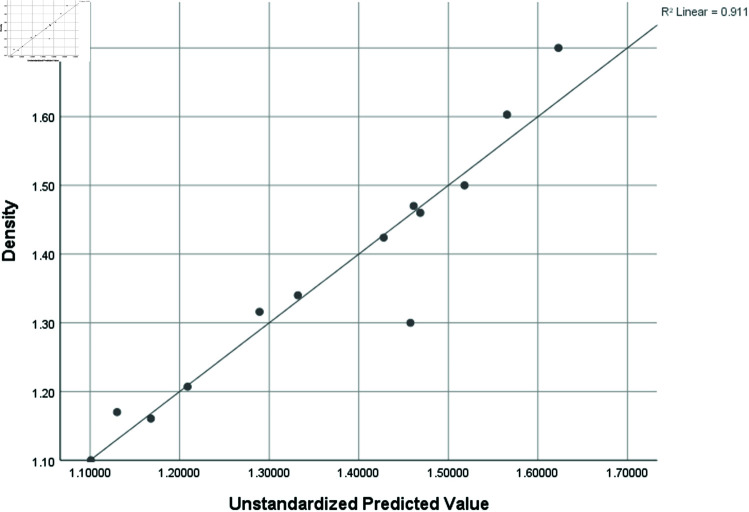
Comparison of linear, quadratic, cubic, logarithmic and exponential regression for Molecular Weight vs $AGO_{2}$.

**Fig 30:**
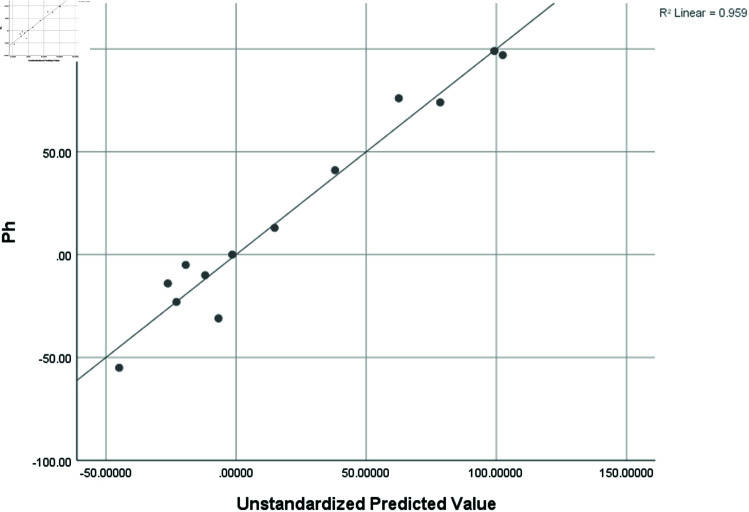
Comparison of linear, quadratic, cubic, logarithmic and exponential regression for Isoelectric Point (pl) vs $AGO_{2}$.

**Fig 31:**
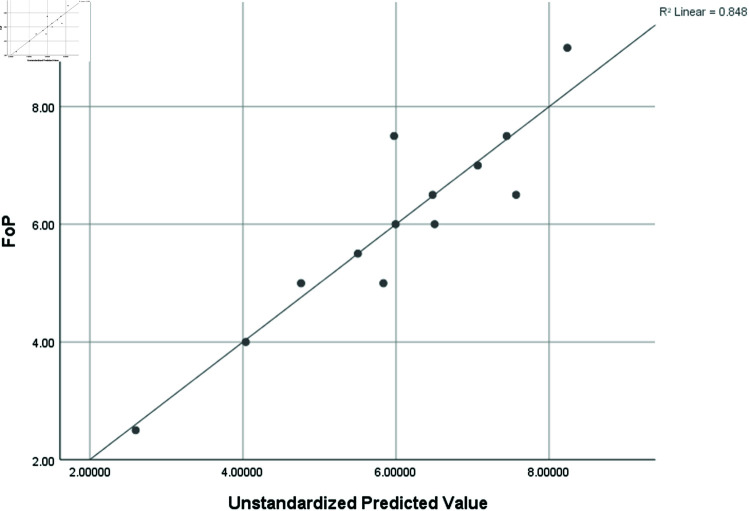
Comparison of linear, quadratic, cubic, logarithmic and exponential regression for Volume (*A*^3^) vs $AGO_{2}$.

**Fig 32:**
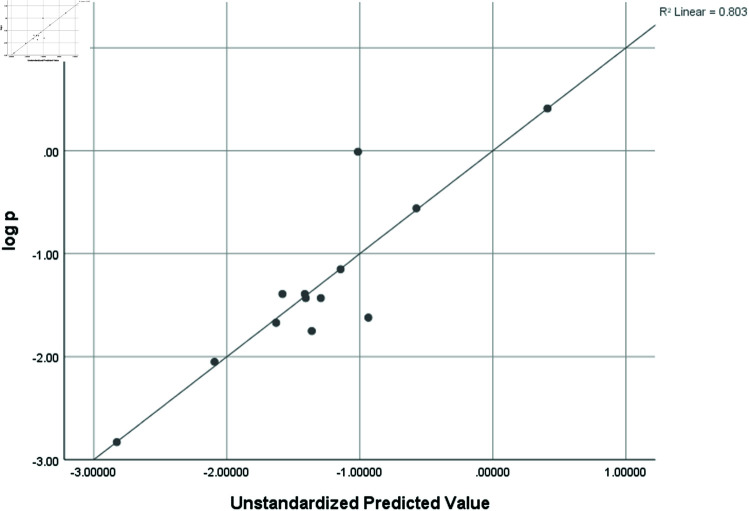
Comparison of linear, quadratic, cubic, logarithmic and exponential regression for Molecular Weight vs $GGO_{1}$.

**Fig 33:**
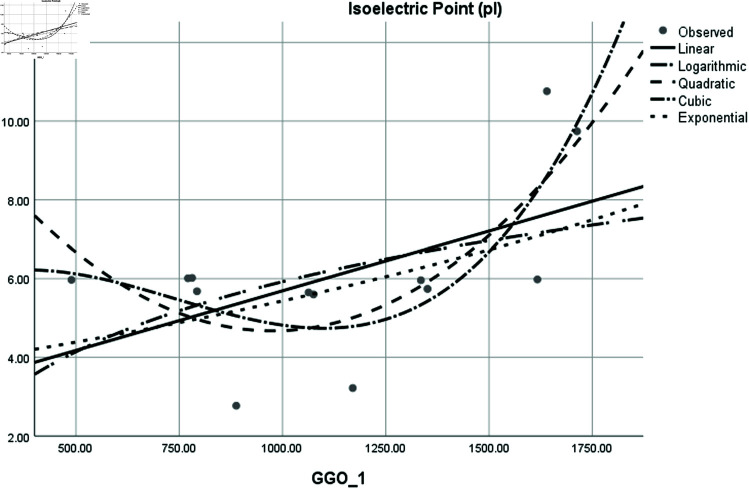
Comparison of linear, quadratic, cubic, logarithmic and exponential regression for Isoelectric Point (pl) vs $GGO_{1}$.

**Fig 34:**
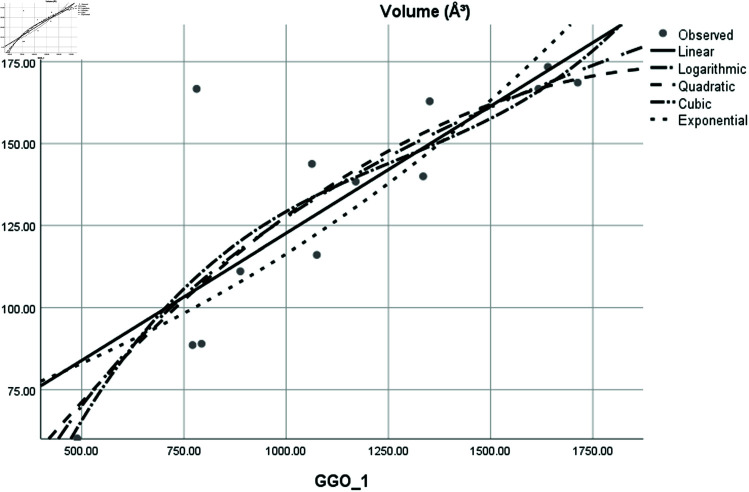
Comparison of linear, quadratic, cubic, logarithmic and exponential regression for Volume (*A*^3^) vs $GGO_{1}$.

**Fig 35:**
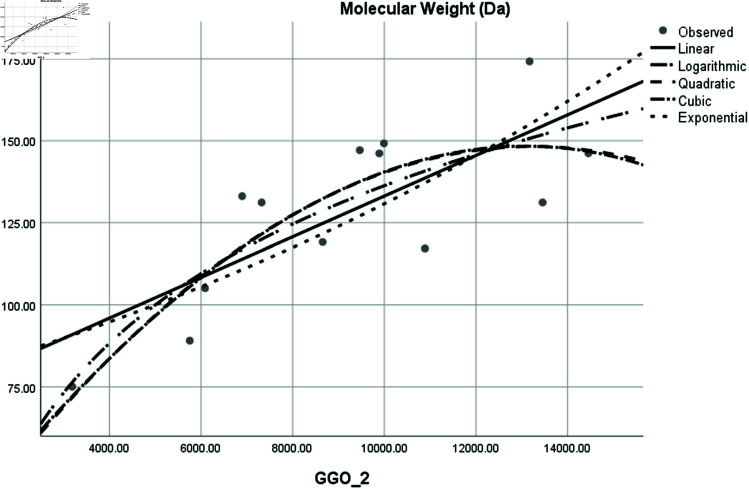
Comparison of linear, quadratic, cubic, logarithmic and exponential regression for Molecular Weight vs $GGO_{2}$.

**Fig 36:**
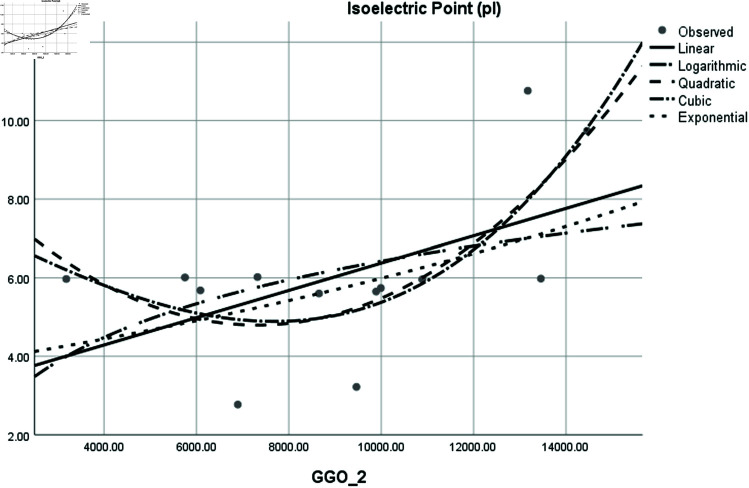
Comparison of linear, quadratic, cubic, logarithmic and exponential regression for Isoelectric Point (pl) vs $GGO_{2}$.

**Fig 37:**
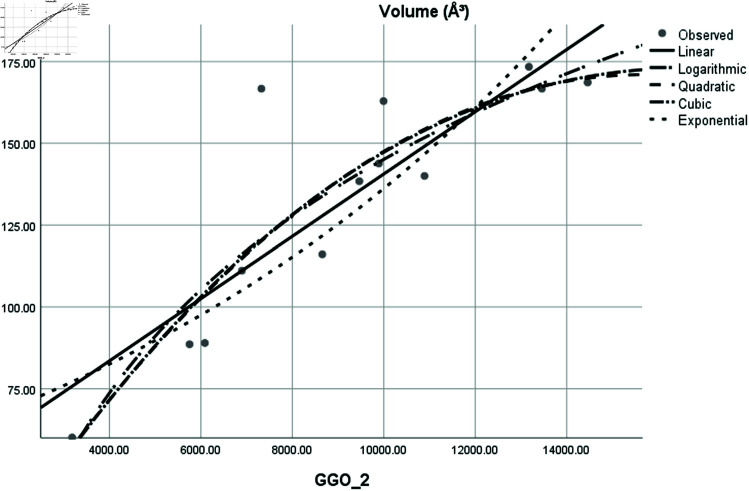
Comparison of linear, quadratic, cubic, logarithmic and exponential regression for Volume (*A*^3^) vs $GGO_{2}$.

**Fig 38:**
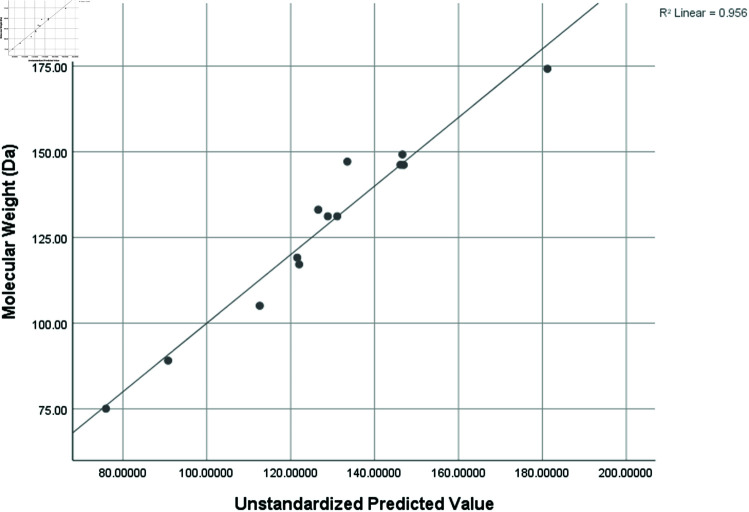
Multiple Regression plots between topological indices $GO_{1}$, $HGO_{1}$, $GO_{2}$, $HGO_{2}$, $AGO_{1}$, $AGO_{2}$, $GGO_{1}$, $GGO_{2}$ and molecular weight.

**Fig 39:**
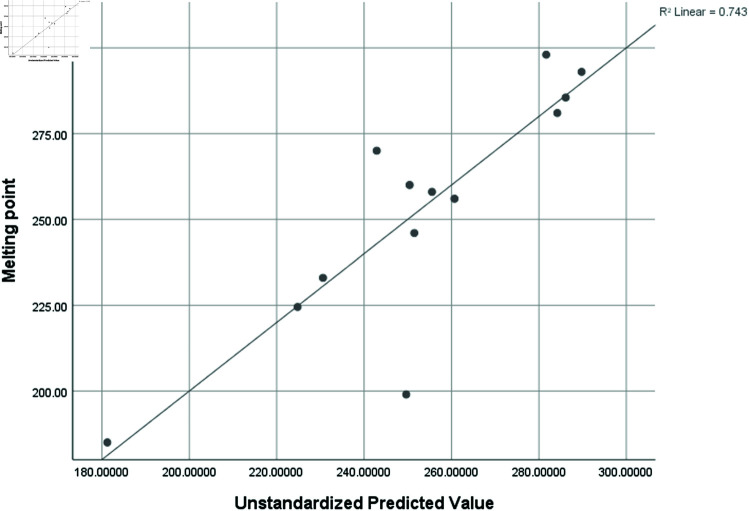
Multiple Regression plots between topological indices $GO_{1}$, $HGO_{1}$, $GO_{2}$, $HGO_{2}$, $AGO_{1}$, $AGO_{2}$, $GGO_{1}$, $GGO_{2}$ and melting point.

**Fig 40:**
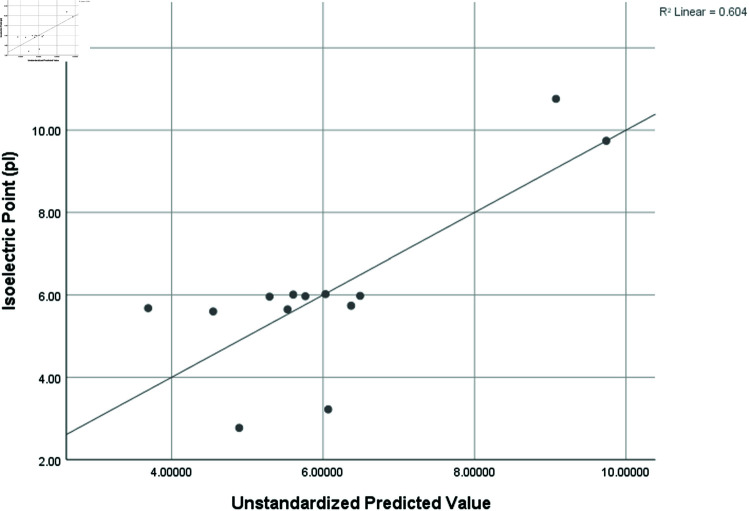
Multiple Regression plots between topological indices $GO_{1}$, $HGO_{1}$, $GO_{2}$, $HGO_{2}$, $AGO_{1}$, $AGO_{2}$, $GGO_{1}$, $GGO_{2}$ and isoelectric point (pl).

**Fig 41:**
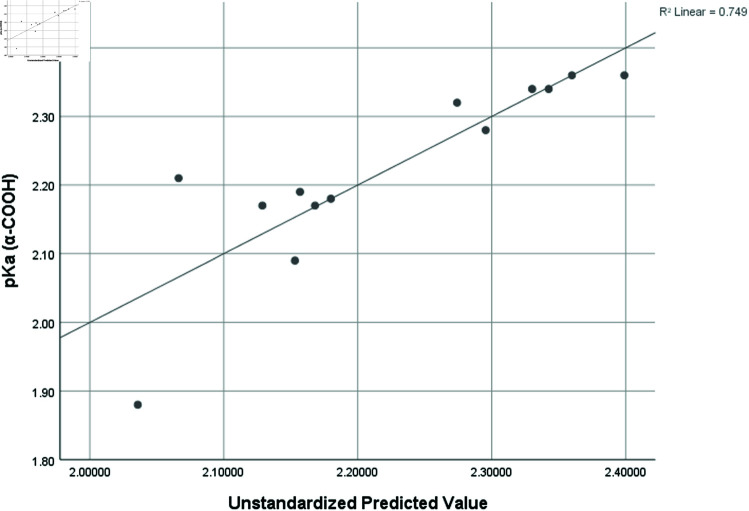
Multiple Regression plots between topological indices $GO_{1}$, $HGO_{1}$, $GO_{2}$, $HGO_{2}$, $AGO_{1}$, $AGO_{2}$, $GGO_{1}$, $GGO_{2}$ and pKa1.

**Fig 42:**
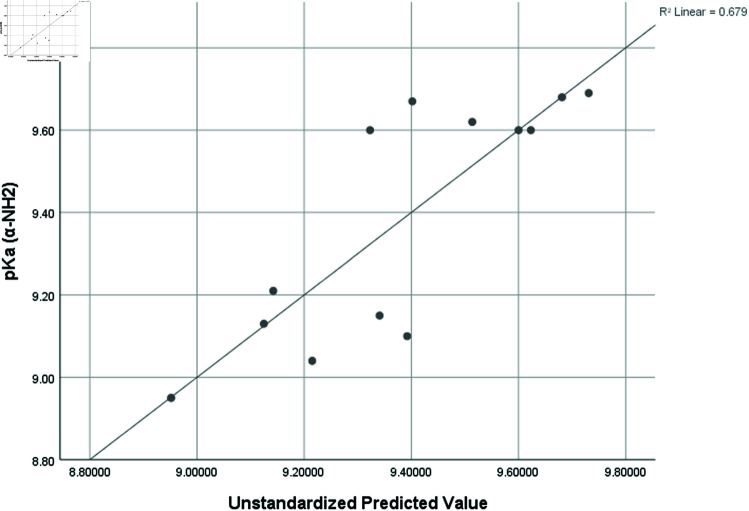
Multiple Regression plots between topological indices $GO_{1}$, $HGO_{1}$, $GO_{2}$, $HGO_{2}$, $AGO_{1}$, $AGO_{2}$, $GGO_{1}$, $GGO_{2}$ and pKa2.

**Fig 43:**
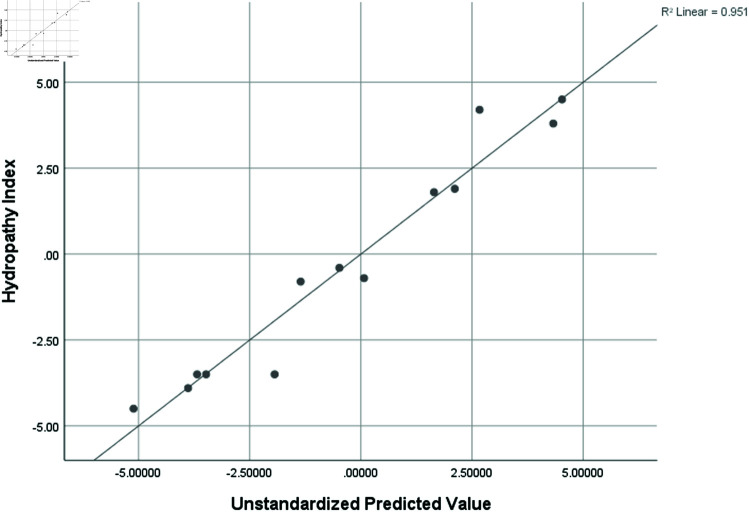
Multiple Regression plots between topological indices $GO_{1}$, $HGO_{1}$, $GO_{2}$, $HGO_{2}$, $AGO_{1}$, $AGO_{2}$, $GGO_{1}$, $GGO_{2}$ and hydropathy index (HI).

**Fig 44:**
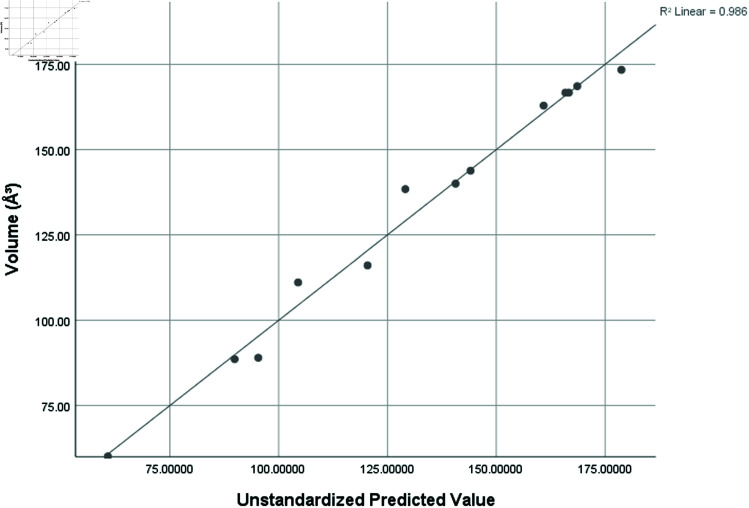
Multiple Regression plots between topological indices $GO_{1}$, $HGO_{1}$, $GO_{2}$, $HGO_{2}$, $AGO_{1}$, $AGO_{2}$, $GGO_{1}$, $GGO_{2}$ and side chain volume (*A*^3^).

**Fig 45:**
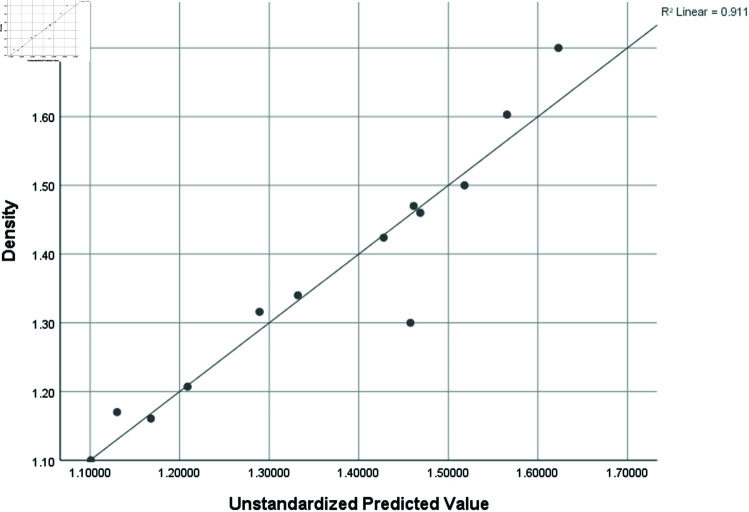
Multiple Regression plots between topological indices $GO_{1}$, $HGO_{1}$, $GO_{2}$, $HGO_{2}$, $AGO_{1}$, $AGO_{2}$, $GGO_{1}$, $GGO_{2}$ and density.

**Fig 46:**
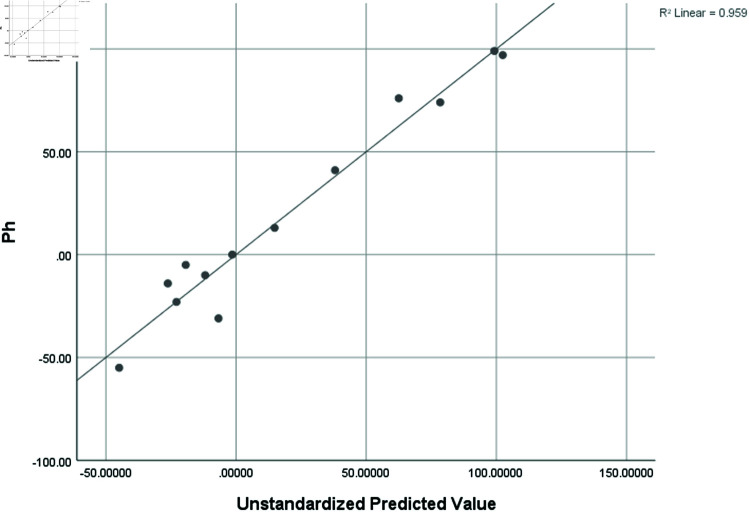
Multiple Regression plots between topological indices $GO_{1}$, $HGO_{1}$, $GO_{2}$, $HGO_{2}$, $AGO_{1}$, $AGO_{2}$, $GGO_{1}$, $GGO_{2}$ and ph.

**Fig 47:**
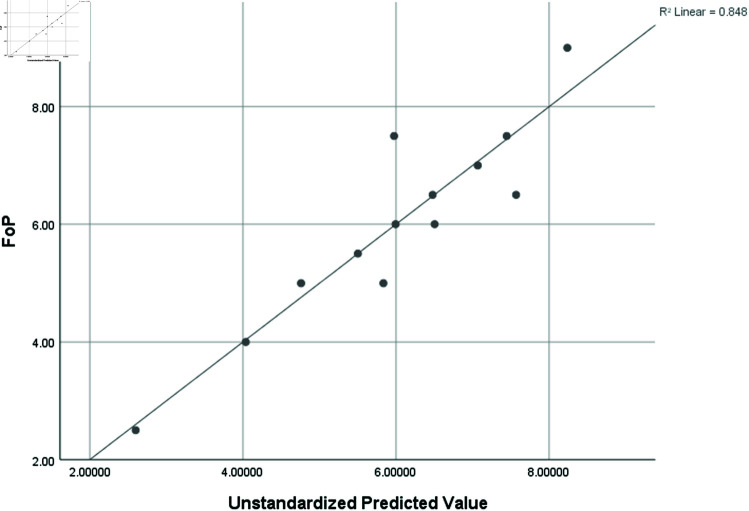
Multiple Regression plots between topological indices $GO_{1}$, $HGO_{1}$, $GO_{2}$, $HGO_{2}$, $AGO_{1}$, $AGO_{2}$, $GGO_{1}$, $GGO_{2}$ and frequency of protenis.

**Fig 48:**
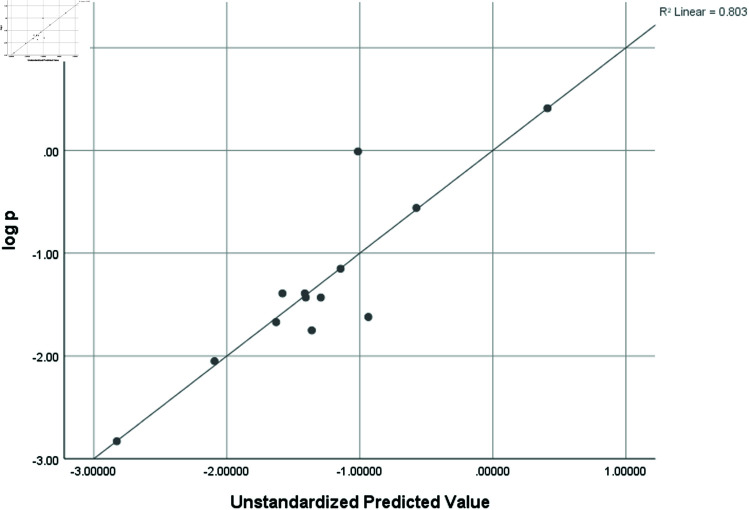
Multiple Regression plots between topological indices $GO_{1}$, $HGO_{1}$, $GO_{2}$, $HGO_{2}$, $AGO_{1}$, $AGO_{2}$, $GGO_{1}$, $GGO_{2}$ and logP.

## Conclusion

The correlations between topological indices and amino acid characteristics were investigated using a variety of regression models, including linear, quadratic, cubic, logarithmic, exponential, and multiple regression. The cubic regression model outperformed the others in terms of capturing intricate nonlinear interactions between the variables, as evidenced by its greatest correlation. Even though the quadratic and linear models produced significant correlations but their slightly lower performance in comparison to the cubic model shows how inadequately simplistic models can capture the complex interactions present in the data set. Reasonable but marginally weaker correlations were found for the exponential and logarithmic models and the cubic model. This implies that there may be nonlinear growth patterns in the relationships between the calculated attributes and topological indices. Despite demonstrating a strong overall correlation with the features, the multiple regression model’s lack of statistical significance (for some) raises the possibility of multicollinearity problems or predictor redundancy. This calls for more research into how the chosen variables interact in order to improve the model. The findings highlight how crucial it is to choose the right regression models when researching the links between structure and properties in biomolecules. The intricate relationships between topological indices and amino acid characteristics are highlighted by the remarkable correlation that the cubic model was able to attain. Rational drug design is largely concerned with the development and optimization of lead compounds, which are the foundational candidates for novel medications. By eliminating the need for lengthy trial-and-error testing, this strategy seeks to improve the efficiency and cost-effectiveness of the drug development process. In order to forecast how a therapeutic molecule will interact with its biological target, it mostly depends on computational methods like molecular modeling.
